# Sustainability diet index: a multi-criteria decision analysis proposal for culinary preparations—A case study

**DOI:** 10.3389/fnut.2025.1586886

**Published:** 2025-07-09

**Authors:** Rodrigo Contreras-Núñez, Andrea Espinoza, Paola Cáceres

**Affiliations:** ^1^Université de Lorraine, Équipe de Recherche sur les Processus Innovatifs (ERPI), Nancy, France; ^2^Program for the Development of Sustainable Production Systems (PDSPS), Faculty of Engineering, University of Santiago of Chile (USACH), Santiago, Chile; ^3^Industrial Engineering Department, Faculty of Engineering, University of Santiago of Chile (USACH), Santiago, Chile; ^4^Department of Nutrition, Faculty of Medicine, University of Chile, Santiago, Chile

**Keywords:** sustainability, index, culinary preparations, menu, MCDA

## Abstract

**Introduction:**

The environmental impact of food production and distribution has driven the need to integrate sustainability into food services. While research has traditionally focused on carbon and water footprints, other key aspects, such as local product consumption, are often overlooked.

**Methods:**

Therefore, this study proposes the development of a sustainability diet index to address these gaps, using Multi-Criteria Decision Analysis. Specifically, the analytical hierarchy process prioritizes sustainability criteria, and the Interactive and Multicriteria Decision Making (TODIM) method ranks them. Two sustainable diet indices are proposed to assess which offers better performance. A case study of a university canteen demonstrates the index's applicability by considering starters, main courses, desserts, and menus.

**Results:**

The results indicate that rankings based on sustainability dimensions provide a broader perspective, highlighting differences among food menus.

**Discussion:**

The index revealed that menus with local and fewer animal-based ingredients score higher in sustainability, underscoring the relevance of environmental and political factors. Future work considers incorporating other cultural traditional food, menu variations, and vegetarian and vegan options.

## 1 Introduction

Currently, the food production sector is one of the main contributors to sustainable development challenges (1, 2), including the depletion of natural resources, the loss of both terrestrial and aquatic biodiversity, and changes in land use. It is noteworthy that 20%–30% of anthropogenic greenhouse gas (GHG) emissions are produced by this sector, with agricultural activities representing the most significant impacts (3, 4). Furthermore, food production demands high freshwater consumption, with agriculture responsible for 70% of total water withdrawals (5, 6). Given the pressure that food systems exert on the environment, various strategies are needed to mitigate the sector's impact, playing an important role in promoting a more sustainable society (7).

The Food and Agriculture Organization of the United Nations (FAO) defines sustainable diets as those with a low environmental impact and contribute to food and nutritional security and healthy life for present and future generations (8). These diets are protective and respectful of biodiversity and ecosystems, culturally acceptable, accessible, economically fair, and affordable; additionally, they must be nutritionally adequate, safe, and healthy, optimizing natural and human resources (8). In summary, sustainable diets consider economic, political, social, environmental, and technological aspects (9–11), and seeks a balance that integrates cultural eating practices, appropriate technological innovations for food systems, and promotes improvements from food production to consumption, including changes in waste management and dietary patterns (12, 13).

In the last decade, the increase in food consumption outside the home has become a global trend (14, 15). This positions massive food services as fundamental actors in the promotion of sustainable food systems (16–19), since small changes in culinary preparations or menus can generate significant impacts when considering that they can cover up to 50,000 preparations (20). However, considering the different aspects of sustainability, with their various measurement forms, offering sustainable diets also becomes a challenge. In this context, sustainability diet indices are required to support decision-makers by performing comprehensive evaluations and allowing for improvements in the sustainability of their offerings, whether culinary preparations or menus.

Therefore, the objective of this study is to develop a multidimensional sustainability diet index applicable at different levels of food disaggregation and adaptable to diverse geographical contexts. Using multi-criteria decision analysis, the index aims to evaluate and rank culinary preparations and menus through a set of weight indicators, thereby supporting evidence-based decision-making in large-scale food services. The applicability and potential of the index are illustrated through a case study, providing practical insights to enhance sustainability practices.

## 2 Related literature

Concerning the related literature, we distinguished three types of food disaggregation analysis: (i) by ingredient, referring to studies focused on the individual analysis of ingredients; (ii) by culinary preparation, referring to food preparations such as desserts; and finally, (iii) by menu, when a set of preparations is analyzed. Most of the related literature studied ingredients individually, while some researchers, such as Engelmann et al. (21) and Cambeses-Franco et al. (22), have assessed culinary preparations, and others, such as Martinez et al. (23), Gómez-Ramos and Rico Gonzalez (24), and Stern et al. (25), have evaluated menus. Nevertheless, among the research assessing more than one food disaggregation, Ernstoff et al. (26) analyzes culinary preparations to evaluate the effects of meat and meatless diets, Castañé and Antón (27) focus on culinary preparations and their environmental impact, and Benedetti et al. (28) compare entire diets, from ingredient production to consumption. These studies collectively emphasize the importance of evaluating food at different levels of disaggregation–from ingredients to menus–to fully understand the environmental impacts of dietary choices.

Furthermore, regarding nutritional aspects, the Nutrient-Rich Food Index (NRF 9.3) is the most used (27, 29), followed by the Healthy Eating Index (HEI) (25, 30), and the Sustainable HEalthy Diet (SHED) (31). However, little research has integrated nutritional aspects into sustainable diet index or indicator assessments. Among those, we can highlight, for example, Luzzani (32) who employs a multi-indicator approach to improve the classification of sustainable dietary patterns, nutritional adaptation, and alignment with local diets, addressing the relationship with the social dimension of sustainability. Similarly, Li et al. (33) develops an indicator system encompassing environmental, economic, social, and nutritional aspects, providing an analytical tool to assess the sustainability of diets from a multi-scale and multidimensional perspective. Additionally, Gazan et al. (34) applies a comprehensive methodology to compile food metrics into a single database, enabling an exhaustive evaluation of food consumption's nutritional, economic, social, and environmental aspects.

The analysis of ingredients individually has traditionally been the main focus. To support massive food service managers transitioning to sustainable food systems, it is essential to consider broader functional units that can help with decision-making, such as culinary preparations or menus. To the best of the authors' knowledge, previous research has not addressed the concept of a sustainable diet while analyzing culinary preparations or menus. Only Hatjiathanassiadou et al. (35) has considered the economic, environmental, and social dimensions without including nutritional aspects, considering all three levels of food disaggregation. Hatjiathanassiadou et al. (35) focuses on how current food systems impact the environment, specifically analyzing the use of environmental footprints—such as carbon, water, and land use footprints—to evaluate the environmental impacts of food consumption while also integrating the economic and social dimensions.

Furthermore, Cáceres et al. (36) presented six indicators validated by experienced chefs and nutritionists in the food services industry. These indicators encompass the impact of ingredient production, such as the carbon footprint (CF) and water footprint (WF), as well as the local origin of ingredients (LAI) (37). They also address recipe composition, including the presence of animal-based ingredients (AI) and red meat (RMI), alongside processing-related aspects like food waste (FW) (36, 37). Despite their validation, these indicators focus mainly on the environmental aspects of sustainability.

Despite their validation, these indicators focus mainly on the environmental aspects of sustainability. This underscores the need for a more comprehensive approach to evaluate diet sustainability, which this study aims to address through the development of an integrative index.

This study is structured as follows. Section 3 describes the proposed sustainability diet index, including identifying and evaluating sustainability indicators and selecting a Multicriteria Decision Analysis (MCDA) tool. Section 4 presents the case study and the findings obtained, including the sustainability rankings of various culinary preparations and menus, followed by Section 5, which discusses the broader implications of these findings. Finally, Section 6 presents the conclusions and future perspectives.

## 3 Materials and methods

This section outlines the methodological framework employed for developing and applying the sustainability diet index (SDI). In this research, two indices are proposed and assessed. The first (*SDI*^1^) considers the nature of sustainability based on its different dimensions, for which a series of indicators is proposed. The second (*SDI*^2^) adopts the indicators proposed by Cáceres et al. (36), which follow the global trend of over-representing the environmental dimension of sustainability and have been validated with relevant stakeholders. Furthermore, sustainability inherently seeks a balance between its different dimensions, but the preferences of the involved stakeholders may vary, so two scenarios are considered for both indices. The first scenario (*s*_1_) corresponds to an equitable prioritization, and the second scenario (*s*_2_) corresponds to a biased one. The latter requires consultation with the involved stakeholders to understand their preferences.

**Figure 1:**
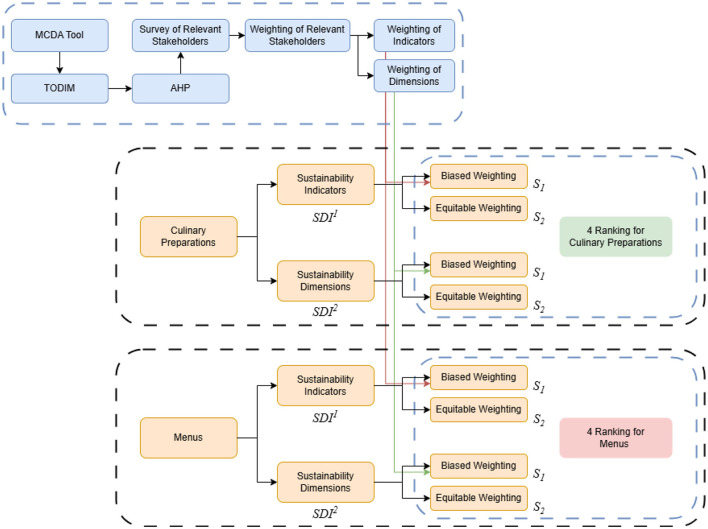
Methodology diagram. MCDA, Multi-Criteria Decision Analysis; TODIM, Interactive and Multicriteria Decision Making; AHP, Analytical Hierarchy Process.

The analysis of these two indices (*SDI*^1^ and *SDI*^2^) in two different scenarios (*s*_1_ and *s*_2_) is carried out to examine whether the different indices generate distinct rankings for the same set of culinary preparations and menus analyzed to ultimately determine which index allows a better differentiation for easier decision-making. Figure 1 presents a schematic diagram of the methodological process used in this study. It shows four rankings for each culinary preparation type, and menus will be compared.

This section presents (1) a sustainability dimensions description for *SDI*^1^; (2) an indicators description for *SDI*^2^; (3) the description of the selected Multi-Criteria Decision Analysis (MCDA) method to establish the preferences of the involved stakeholders to generate the required data for evaluating the index in the biased prioritization scenario; and (4) a general description of the data collected for the set of culinary preparations and menus to be analyzed.

### 3.1 Sustainability dimensions description for *SDI*^1^

While sustainability has historically been defined as the balance between social, economic, and environmental aspects, technological and political considerations should not be overlooked. For example, the complexity of culinary preparation may discourage food services from implementing certain dishes due to the need for a wide variety of ingredients, lengthy preparation times, specialized skills, or additional equipment. Meanwhile, governmental factors are particularly relevant in large-scale food services that rely on government organizations, where policies can be tested for efficacy before being introduced to the general public. Thus, economic, social, environmental, political, and technological components are essential for a comprehensive understanding of sustainability (11, 38).

Two aspects of the economic dimension are addressed. First, resource scarcity is estimated in monetary terms through Life Cycle Assessment (LCA), using the “USD2013” indicator from the ReCiPe Endpoint method, which reflects the costs associated with the extraction of minerals and fossils (39). Second, the costs related to food acquisition are analyzed, providing a comprehensive view of the economic impact of sustainable eating practices. These indicators were chosen due to their ability to facilitate an understanding of the actual costs associated with sustainability.

The social dimension focuses on two aspects. First, it quantifies Disability-Adjusted Life Years (DALY), which reflect losses associated with diseases, disabilities, or premature deaths, using the DALY indicator of the ReCiPe Endpoint method (39). Additionally, the nutritional quality of foods is evaluated through the Nutrient-Rich Foods Index (NRF 9.3) (40), measuring the nutritional contribution of foods in the diet. Including these indicators allows for a comprehensive assessment of social and nutritional impacts, addressing public health and diet quality.

Regarding the environmental dimension, impact categories such as Global Warming Potential (GWP), based on the Intergovernmental Panel on Climate Change (IPCC) methodology (41), and water consumption, measured through the Available WAter REmaining (AWARE) method (42), are analyzed. Additionally, species loss over time will be assessed to determine the impact on biodiversity and ecosystems using the “Species.yr" indicator of the ReCiPe Endpoint method (39). These tools provide a comprehensive and easily interpretable evaluation of environmental impacts. This selection ensured a thorough and relevant assessment of the overall environmental impact.

Concerning the political dimension, the focus is on promoting local consumption and production by evaluating the percentage of locally sourced foods (36). This measure aims to strengthen regional economies and increase locally generated jobs. The choice of this indicator is justified by its relevance in promoting sustainable food policies and its direct impact on local economies.

On the other hand, the technological dimension will be analyzed by considering the number of ingredients used in each recipe based on the assumption that a greater number of ingredients may require longer processing times. This is established as an operational limitation. This approach seeks to optimize ingredient efficiency and promote more sustainable practices in the kitchen. Furthermore, it allows for identifying opportunities to improve technological efficiency in food preparation.

### 3.2 Indicators description for *SDI*^2^

The CF is an environmental indicator used to quantify the greenhouse gas (GHG) emissions produced directly and indirectly throughout a production system, following the principles of ISO 14064 for LCA. This environmental indicator is considered the most common in evaluating eating habits (7, 43). However, Nemecek et al. (14) notes that considering GHG emissions alone is insufficient to address the environmental impacts of food, as it lacks other impacts related to agricultural production and water consumption. Therefore, it is also necessary to evaluate food's water footprint (WF). The water needs in food production systems for crop irrigation and food processing have received particular attention in recent years due to the decreasing availability of water worldwide (44). Thus, it is concluded that it is important to seek the reduction of both environmental impacts when evaluating a sustainable diet.

On the other hand, it is relevant to promote the consumption of local foods (LAI), avoiding the consumption of imported foods. Mainly because imported foods require more processing, packaging, and travel long distances, generating a significant environmental impact (45). Additionally, referring to local foods commonly alludes to seasonal foods, which are produced under minimal artificial intervention in crops, thereby contributing to the reduction of environmental impact and fostering local employment generation (7, 45, 46). Ultimately, local production must be considered from an environmental and social perspective when discussing sustainable diets.

Furthermore, according to González-García et al. (47), animal-based ingredients (AI) have the highest scores in terms of CF and WF, especially red meat (RMI) (48). Therefore, if the goal is to reduce their impact on diets from a sustainable perspective, it is important to consider reducing meat portion sizes, decreasing frequency, or adopting new dietary guidelines (49, 50). However, this can lead to potential malnutrition and undernutrition problems (49, 51). Thus, studying the balanced incorporation of animal-based ingredients is essential for a sustainable diet.

Lastly, reducing food waste (FW) decreases the environmental burden on landfills, reduces the costs associated with its management, and promotes more efficient use of available resources. This aligns with sustainability and environmental responsibility (45, 52).

### 3.3 Selection of Multi-Criteria Decision Analysis (MCDA)

To construct the *SDI*, two weighting assignment scenarios will be analyzed. The first scenario, *s*_1_, corresponds to an equal weighting for all criteria, while the second scenario, *s*_2_, corresponds to a biased weighting assigned by nutrition experts. This comparison allows us to analyze whether there are differences between the rankings' compositions to understand the impact of the preferences.

To select an MCDA method that allows for the assignment of a biased weighting, the Multiple Criteria Decision Analysis Methods Selection Software (MCDA-MSS) created by Cinelli et al. (53) was used. This software includes a set of more than 200 MCDA methods, representing different schools, approaches, and trends within MCDA. The tool is valuable for selecting and identifying possible errors when choosing the MCDA method. It works through a series of questions aimed at helping analysts and researchers understand and describe complex decision-making processes, facilitating the recommendation and selection of the most appropriate MCDA methods for each case study (53).

For this study, the MCDA-MSS software recommended the use of the TODIM method, an acronym in Portuguese for Interactive and Multicriteria Decision Making (54, 55), based on Prospect Theory (56). This approach allows for ranking alternatives according to the preferences of decision-makers, where evaluations are not solely based on the final result but incorporate a model that reflects their perception of gains and losses relative to a reference point (55, 57). Compared to other MCDA techniques that typically assume linear and compensatory reasoning, TODIM introduces a more behaviorally realistic model, enhancing its applicability to multidimensional evaluations (55). This is particularly valuable in sustainability assessments, where decision-making often involves conflicting objectives. One of the distinguishing features of the TODIM method is its ability to address uncertainty, a relevant characteristic in sustainability-related decision-making (58). Additionally, this method is notable for performing pairwise comparisons between decision criteria, offering simple yet effective resources to resolve potential inconsistencies arising from these comparisons. Thus, it allows for constructing a criteria hierarchy and considering interdependence relationships between alternatives (59).

According to the study (60), the TODIM method consists of the following steps:

1. **Evaluation of alternatives according to criteria:** All alternatives are evaluated based on the previously defined criteria. An evaluation matrix with all necessary data in numerical form is created.
2. **Normalization of the evaluation matrix:** The data in the evaluation matrix are normalized by dividing the value of each alternative by the total sum of values assigned to all alternatives for that criterion. This normalization generates a new matrix with values from 0 to 1, ensuring a direct comparison between them.
3. **Definition of criteria weighting:** The weights of each criterion are determined and normalized to calculate a dominance matrix. The decision-makers must indicate the reference criterion (the most important) for the calculations. The weight of each criterion, determined by the decision-makers on a numerical scale, is normalized by dividing the weight of each criterion by the weight of the reference criterion, thus generating the normalized weights *W*_rc_, where *W*_rc_ is the weight of criterion *c* divided by the weight of the reference criterion *r*. To determine the weights of each criterion, they must be prioritized through a mutual comparison. This process is carried out using the Analytical Hierarchy Process (AHP) method (61), which allows for a comparison between different study criteria to determine which is preferred. For this, a rating scale is used, ranging from 1 to 9, where:

- (1) Equal importance: Both criteria contribute equally.
- (3) Moderate importance: Judgment slightly favors one criterion over the other.
- (5) Strong importance: One criterion is clearly more important than the other.
- (7) Very strong importance: One criterion strongly dominates the other. This dominance is proven.
- (9) Extreme importance: One criterion dominates the other with the highest possible order of magnitude.
- (2,4,6,8) Intermediate values: Values used to express preferences between two values of the above scale.

After all the comparisons, the weighting assigned by the nutrition professionals to each criterion is indirectly calculated, providing a quantitative measure of its importance. Additionally, the consistency of the responses is verified using the Consistency Index (CI) and the Consistency Ratio (CR). The CR must be less than or equal to 0.10 (10%) to ensure the study's validity and the reliability of the derived decisions. A CR value above this threshold indicates the presence of significant inconsistencies in the pairwise comparisons, which may require a review and correction of the evaluations to ensure consistency. Finally, the individual assignments of each decision-maker are averaged to determine the overall results, thus ensuring an accurate and well-founded evaluation.

**4. Degree of dominance of the alternative:** A mathematical procedure must be followed to determine the degree of dominance between alternatives, based on Prospect Theory (56). The dominance of alternative *A*_*i*_ over another alternative *A*_*j*_ is calculated using Equation 1. This is given by the sum of relative gains and losses between the alternatives. Equation 2a describes the gain part of the value function, while Equation 2c represents the loss part. Equation 2b is applied when neither gain nor loss exists.

(1) $$\begin{array}{l} \delta \left(A_{i},A_{j}\right)=\overset{m}{\underset{c=1}{\sum }}{\text{Φ}}_{c}\left(A_{i},A_{j}\right),\forall \left(i,j\right). \end{array}$$

When:


(2) $${\text{Φ}}_{c}(A_{i};A_{j})=\{\begin{array}{ll} \sqrt{\frac{w_{rc}(P_{ic}-P_{jc})}{\sum_{c=1}^{m}w_{rc}}} & \text{si, }(P_{ic}-P_{jc})>0,\;(a)\\ 0 & \text{si, }(P_{ic}-P_{jc})=0,\;(b)\\ -\frac{1}{\theta }\sqrt{\frac{\left(\sum_{c=1}^{m}w_{rc})(P_{jc}-P_{ic}\right)}{w_{rc}}} & \text{si, }(P_{ic}-P_{jc})<0.\;(c) \end{array}$$

Where:

- Φ_*c*_(*A*_*i*_, *A*_*j*_) represents the measure of the dominance of *A*_*i*_ over *A*_*j*_.
- *m* indicates the total number of criteria evaluated.
- *c* refers to a specific criterion, with *c* = 1, …, *m*.
- *w*_*rc*_ is equal to *w*_*c*_ divided by *w*_*r*_ where *r* is the reference criterion.
- *P*_*ic*_ and *P*_*jc*_ are the performances of alternatives *A*_*i*_ and *A*_*j*_ in relation to *c*.
- θ is the attenuation factor of losses, affecting the shape of the value function in loss situations.

This structure allows for evaluating the alternatives, considering both gains and losses concerning the established criteria, reflecting the complexity of decisions in uncertain environments, and providing a basis for informed decision-making.

5. **Global dominance degree:** Once all the dominance matrices for each criterion are calculated, a final dominance matrix is created, where δ(*A*_*i*_, *A*_*j*_) sums all the corresponding elements of the partial matrices. Then, to quantify the overall value of each alternative *i*, Equation 3 is used. This equation normalizes all dominance measures, allowing for a ranking of each alternative based on their values. Finally, the result is a complete ordering of all the alternatives, providing an analysis of all available options for decision-making, thereby facilitating the identification of the most sustainable options.

(3) $$\begin{array}{l} \xi_{i}=\frac{\sum_{j=1}^{n}\delta \left(A_{i},A_{j}\right)-\min_{j=1}^{n}\delta \left(A_{i},A_{j}\right)}{\max_{j=1}^{n}\delta \left(A_{i},A_{j}\right)-\min_{j=1}^{n}\delta \left(A_{i},A_{j}\right)}. \end{array}$$

### 3.4 Data collection

In Chile, food and nutritional security presents significant challenges. According to the report (62), the population over 15 years old in the country ranks second in Latin America for the highest rates of overweight and obesity, as well as second in the consumption of ultra-processed foods in the region (63). Additionally, it is among the top 10 countries with the highest per capita meat consumption worldwide (64). This reality highlights the importance of assessing the environmental impact of the Chilean diet and implementing improvements that promote sustainability. In response to this situation, Chile has developed the National Sovereignty Strategy for Food Security (65), establishing a comprehensive approach to the food system to guarantee the right to food. Faced with both global and national challenges, such as the dependence on imported foods and the degradation of natural resources, this initiative focuses on promoting access to productive resources, fostering sustainable practices, valuing local production, and ensuring the conservation of natural resources and biodiversity, thus promoting a more sustainable food system.

In this context, this research focused on a public university in Chile, located in Santiago. The university has a strategic sustainability objective, evidenced through the annual publication of a sustainability report. Unlike other higher education institutions that opt to outsource food services, this university directly manages its cafeteria. In practice, the university cafeteria is managed by a specific operational area, capable of offering 3,000 culinary preparations per day, highlighting its capacity to significantly influence the promotion of sustainable eating practices within the university community.

The main data used in this study are derived from the ingredients used in culinary preparations. Data collection was collected through interviews with the cafeteria manager and through collaborative work with kitchen staff and institutional nutritionists. Using daily menus as a reference, the team reconstructed and validated the quantity of each ingredient used per preparation, ensuring accurate measurements based on standard serving practices. This collaborative process allowed for a detailed and context-specific dataset. In total, meals offered over a four-month period were analyzed, providing a comprehensive understanding of the food composition available to students and staff.

To evaluate the sustainability dimensions and indicators such as carbon footprint, water footprint, impact on biodiversity and ecosystems, resource scarcity in monetary terms, and disability-adjusted life years, the software SimaPro version 9.4.0.4 was used. Specialized databases such as Ecoinvent 3, Agribalyse_V3.01, Agri-footprint 5, Exiobase, World Food LCA, and WEEE LCI were utilized. This allowed for precise estimates for each ingredient studied, ensuring an accurate analysis of the culinary preparations. Various factors were considered for the comprehensive analysis of the emissions generated by the culinary preparations. Initially, the emissions originating from food production and the emissions resulting from the transportation of each ingredient from its production site to the preparation site in the university were evaluated based on the study (38). The food production locations were identified through extensive research, primarily using data from the Chilean Office of Agricultural Studies and Policies (ODEPA) (66, 67). Additionally, the amount of waste generated by each ingredient during culinary preparation was estimated based on information collected by Cáceres Rodríguez and Lataste Quintana (68). Lastly, the emissions associated with the transportation of food waste to the Santa Marta landfill in the Metropolitan Region of Chile, including the emissions related to the landfill operation, were incorporated.

A price search was conducted to determine the costs associated with acquiring ingredients for culinary preparations, expressed in Chilean Pesos (CLP). Most of these data were obtained from ODEPA (69). Ingredients not found in the ODEPA database were searched for in the public market, given its relevance as a procurement platform for public sector entities (70). Traditional supermarkets were consulted (71, 72) for information on ingredients absent from these sources. With all the collected price information, the total value of each culinary preparation was calculated, considering the amount of each ingredient used.

To determine the nutritional information of each ingredient, the USDA Food and Nutrient Database was used (73). The daily reference values and maximum recommended values for the nutrients used in this process were adopted from the study by Drewnowski (40). This information allowed for evaluating the nutritional quality of each culinary preparation. Additionally, each ingredient was classified according to its food category.

## 4 Results

This section presents the results obtained for the sustainability diet index for culinary preparations and menus, considering the case study of the University of Santiago of Chile (USACH) for both defined scenarios.

### 4.1 Data collection of the case study

The data collection for the case study is focused on the culinary preparations offered by the university cafeteria, which are organized into menus that include a starter, a main course, and a dessert, i.e., three culinary preparations. According to the information collected in García-Leal et al. (38) and supplemented by recent interviews with the cafeteria managers, 56 different starters, 92 main courses, and 33 desserts were identified. For the menu analysis, we focused on those effectively delivered to the community and presented combinations of the identified culinary preparations, totaling 67 menus. This allows for a varied analysis of food options from a sustainability perspective. The detailed values of the criteria for each preparation can be found in the Supplementary material.

### 4.2 Survey analysis

Implementing the TODIM method allows for weighting the criteria under study (*s*_2_). In this particular case, two different analyses are considered: one focused on sustainability dimensions (*SDI*^1^) and the other on indicators (*SDI*^2^). To establish these weightings, surveys were conducted with nutrition experts to identify the aspects they consider most relevant in sustainable food. The surveys were based on the AHP criteria ranking scale (61). To ensure the representativeness of the sample of experts, the existence of approximately 12,400 nutritionists in Chile was considered, according to the study by Durán Agüero et al. (74). Establishing a confidence level of 85% and a margin of error of 15%, it was calculated that at least 23 surveys were needed to obtain a significant sample.

The survey was designed in an online format and validated by the ethics committee of the University of Santiago de Chile (75). Invitations were sent via email, and promotion was done through social media, specifically Instagram. Each invitation included informed consent detailing relevant study information and ensuring participant confidentiality to guarantee research transparency. Between October and December 2023, 25 experts with diverse professional profiles participated. These included: 17 experts in research and teaching (focused on scientific research related to nutrition); 2 clinical experts (focused on the treatment and prevention of diseases through nutrition); 1 sports expert (dedicated to helping individuals who engage in intense physical activity); 1 expert in primary and community care (focused on public health and nutritional education programs in communities); and four food service experts (specialized in planning and managing food services). These experts provided their perspectives on the different sustainability dimensions and indicators, providing the weights presented in Tables 1, 2, respectively.

**Table 1:** Biased weighting of sustainability dimensions according to experts (*SDI*^1^–*s*_2_).

**Dimensions**	**Research**	**Clinical**	**Sports**	**Primary and community care**	**Food service**	**Global**
Economic	0.068	0.031	0.075	0.348	0.191	0.142
Environmental	0.281	0.477	0.510	0.183	0.375	0.365
Social	0.235	0.250	0.254	0.341	0.255	0.267
Political	0.248	0.158	0.111	0.052	0.093	0.132
Technological	0.169	0.083	0.050	0.077	0.085	0.093
Consistency index	0.057	0.103	0.099	0.108	0.110	-
Consistency ratio	0.051	0.092	0.088	0.097	0.098	-

**Table 2:** Biased weighting of indicators according to experts (*SDI*^2^–*s*_2_).

**Indicators**	**Research**	**Clinical**	**Sports**	**Primary and community care**	**Food service**	**Global**
Carbon footprint	0.099	0.177	0.301	0.088	0.111	0.155
Water footprint	0.172	0.219	0.273	0.071	0.274	0.202
Local ingredients	0.151	0.139	0.168	0.295	0.090	0.169
Animal-based ingredients	0.081	0.127	0.035	0.318	0.168	0.146
Red meat (beef) ingredients	0.159	0.137	0.030	0.151	0.247	0.145
Food waste	0.338	0.201	0.193	0.078	0.112	0.184
Consistency index	0.023	0.122	0.121	0.125	0.088	-
Consistency ratio	0.018	0.098	0.098	0.100	0.071	-

The analysis of the CI and CR indicates a satisfactory level of coherence among the experts' evaluations, with consistency ratios below 10%, demonstrating a high degree of agreement in their judgments. Notably, all consistency ratios are below 0.10, except for one that exactly reaches this threshold. This level of consistency reflects the reliability of the assigned weightings, indicating that the experts' evaluations are logically coherent and can be considered solid for decision-making based on sustainability criteria.

Regarding the weightings assigned to the sustainability dimensions, Table 1 illustrates that the environmental dimension stands out. This highlights the experts' recognition of the urgent need for sustainable actions to protect the environment. Across all specialties, the social dimension is highly valued, reflecting a shared interest in promoting collective wellbeing and social responsibility. Moreover, the balanced weighting among the environmental, social, and political dimensions, as noted by researchers, clinicians, and sports nutritionists, suggests a comprehensive perspective on sustainability. Food service and community care professionals emphasize the classic dimensions of sustainability, which aligns with the practical applications of their work. Although the technological dimension receives comparatively less attention, it is recognized as a promising area for optimizing sustainability within the food chain.

Analyzing the weightings provided by global experts concerning the various indicators, it becomes evident that the water footprint holds the greatest significance (see Table 2). This is particularly relevant given Chile's water crisis, as the country ranks 18th for “high water stress” according to a recent report (76). Additionally, food waste is identified as a major cost factor, affecting both energy usage and financial resources, and it has a considerable environmental impact on Chilean households (77). These indicators demonstrate a commitment to improving water management efficiency and reducing waste. In contrast, animal-based ingredients and red meats received the lowest weightings, indicating they are considered less of a priority in this context.

The analysis reveals variations in priorities based on the experts' specializations. Researchers and educators strongly emphasize water and waste management, reflecting their focus on long-term sustainability in response to Chile's water crisis. Clinicians and nutritionists working in food services also underscore the importance of the water footprint, aligning their priorities with disease prevention and efficient water use goals. In contrast, sports experts highlight the carbon footprint, underscoring the implications of climate change in the sports sector. Finally, community nutrition specialists emphasize the consumption of local and animal-based ingredients, reflecting the importance of sustainable nutrition practices within communities.

### 4.3 SDI results for culinary preparations and menus in both scenarios

**Figure 2:**
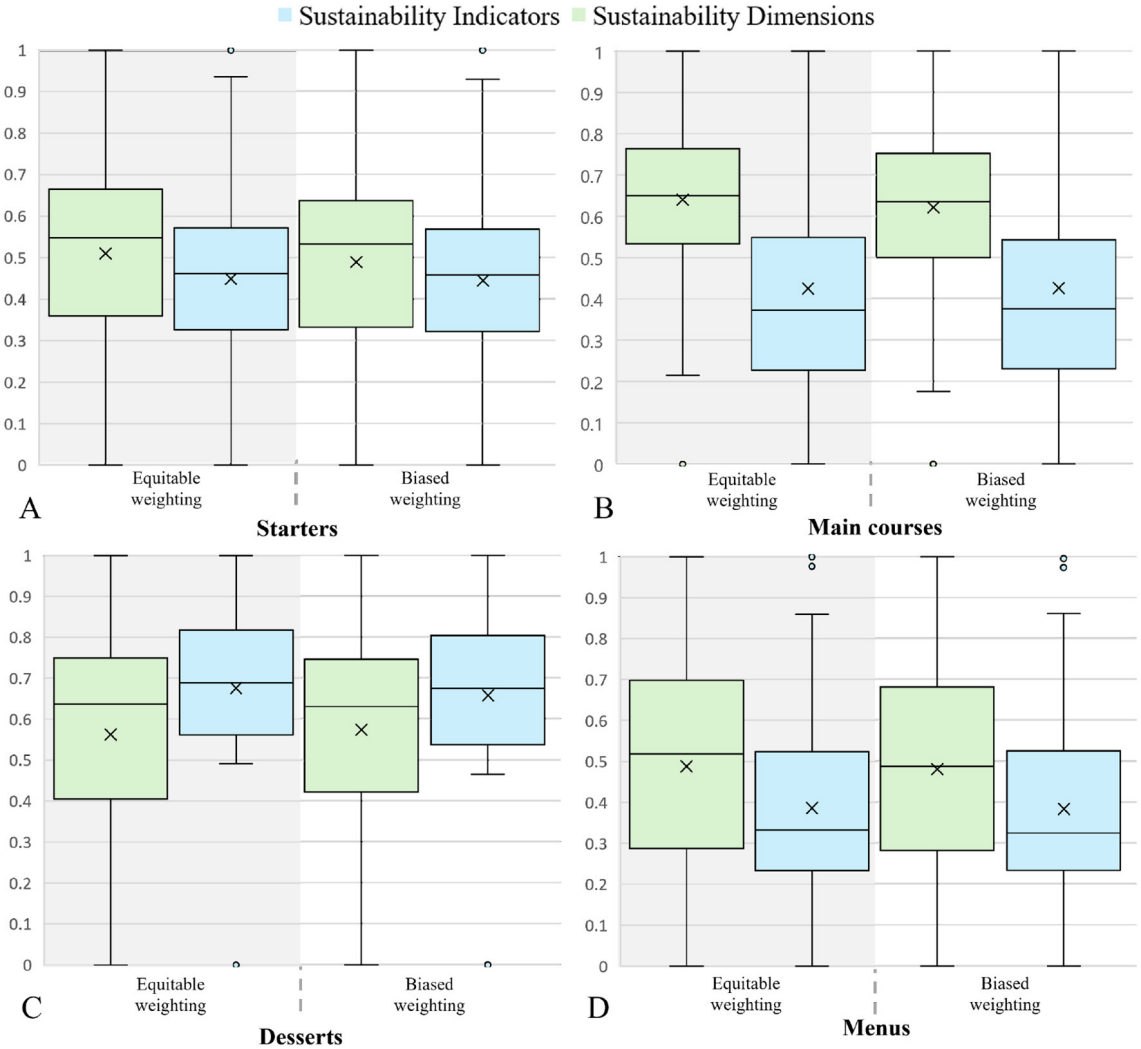
Box plots. Rankings of culinary preparations and menus with sustainability dimensions and indicators (*SDI*^1^ and *SDI*^2^). **(A)** Starters. **(B)** Main courses. **(C)** Desserts. **(D)** Menus.

To conduct a more detailed analysis, the results were separated by food aggregation level: (i) culinary preparations (including starters, main courses, and desserts) and (ii) menus. Then, both the index (*SDI*^1^ and *SDI*^2^) and scenarios (*s*_1_ and *s*_2_) are assessed by each category. Figure 2 shows the variance assessment for the rankings obtained in each case.

Figure 2A shows the results of the four rankings for starters. Considering the rankings by sustainability dimensions for starters (*SDI*^1^), we observe greater variability than the indicator rankings. This variability is indicated by a standard deviation of 0.2174 for the dimensions compared to 0.2160 for the indicators. Despite this variability, no significant differences are noted between the results obtained using equitable and biased weights (*s*_1_ and *s*_2_). However, a clear preference remains for recipes that use vegetables and local products. Notable examples include “Spinach with carrots” and “Broccoli with cauliflower” which stand out for their reduced environmental footprint, ranking among the top ten in terms of economic and social dimensions. Conversely, meat-based recipes are classified as less sustainable and occupy the lowest positions in the dimensions rankings (see Supplementary Table S8). The results show no significant differences in the rankings for the indicators (*SDI*^2^) regardless of the type of weighting applied, whether equitable or biased (*s*_1_ and *s*_2_). It is noted that starters with a higher inclusion of plant-based and local ingredients, such as “Cabbage with olives,” “Vegetable cream,” and “Spinach with carrots,” stand out for their sustainability in (*SDI*^2^). In contrast, preparations that include meat, such as “Beef consomme” and “Empanada de pino,” are positioned lower, indicating a more significant negative environmental impact due to their high carbon footprints and the use of red meat ingredients. At the lower end of both indicator rankings, for starters, preparations such as “Celery with avocado and vegetables,” “Tomato with coriander,” and “Celery with bell pepper” are characterized by their considerable water footprint and significant waste generation. Additionally, Figure 2A show that (*SDI*^2^) presents a narrower interquartile range (IQR) compared to the sustainability dimensions. This indicates less dispersion in the evaluation of the recipes.

Regarding the analysis of the rankings for main courses, presented in Figure 2B, preparations predominantly composed of vegetables and legumes, such as “Pasta with mushroom and corn sauce,” “Noodles with sauce and vegetables,” and “Humitas” rank highest in both indicator rankings. This suggests that the balanced integration of plant-based ingredients significantly contributes to the sustainability of the culinary preparation, mitigating the negative impacts of animal-based ingredients. Nonetheless, some culinary preparations that include meat and are found in the first quartile of the rankings seem to benefit from the significant inclusion of vegetables, legumes, or grains, partially offsetting the negative impact of meat. On the other hand, dishes with a high content of animal ingredients, such as “Roast beef with mashed potatoes,” “Roast pork with mixed vegetables,” and “Loin with corn pie,” are among the least sustainable. They face challenges not only in terms of carbon footprint and water consumption, negatively characterizing the environmental dimension, but also involve higher costs, negatively affecting the economic dimension (see Supplementary Table S9). It is worth mentioning that, in general, predominantly vegetable dishes tend to be associated with lower acquisition costs, highlighting their cost-benefit advantage from a sustainability perspective. Furthermore, examining the box plots of the main courses, a reduced variability in sustainability dimensions is revealed compared to individual indicators. This lower variability is reflected in a standard deviation of 0.1883 for sustainability dimensions and 0.2311 for indicators. This suggests a more uniform evaluation of sustainability, regardless of the specific weights applied, indicating a stronger consensus on the overall sustainability of these culinary preparations.

Figure 2C allows the analysis of the rankings of preparations categorized as desserts. When evaluating the rankings from different sustainability dimensions (*SDI*^1^), it is noted that desserts with multiple ingredients and more complex preparation processes tend to be classified as less sustainable, negatively impacting the technological dimension. These include “Chocolate flan,” “Fried bananas with cream,” and “Carrot cake,” which are also characterized by offering lower nutritional value and being associated with higher costs compared to the desserts under study. Despite the need to prepare “Pineapple jelly,” it remains the most sustainable dessert. This suggests that its simplicity in both preparation and ingredients, along with the absence of waste generation, leads to a lower impact on the sustainability dimensions (see Supplementary Table S10). According to the indicators ranking, the most sustainable desserts are “Pineapple jelly,” “Baked milk,” and “Brazo de reina,” standing out under both weightings. These desserts are notable for their limited environmental impact, evidenced by low carbon and water footprints. “Baked milk” and “Brazo de reina” particularly stand out for their use of local ingredients, contributing to mitigating their environmental impact compared to other desserts that do not incorporate these ingredients. In contrast, desserts such as “Mango” and “Raspberry” rank among the least sustainable, primarily due to their high environmental footprints. The box plots in Figure 2C indicate that desserts, in terms of indicators, show a medium-high sustainability rating and demonstrate comparatively lower variability than the evaluation by sustainability dimensions. This is reflected in a variance of 0.0356 and a standard deviation of 0.1888 for the indicators, compared to a variance of 0.0662 and a standard deviation of 0.2573 for the dimensions.

Figure 2D presents the results for the four rankings obtained for the menus. Regarding the sustainability dimensions rankings, the menus identified as the most sustainable include n°58: “Lettuce with green beans and carrot,” “Vegetarian charquican,” and “Orange”; n°1: “Lettuce with carrot,” “Lentils,” and “Orange;” and n°10: “Cabbage with mixed lettuces,” “Vegetarian potato pie,” and “Orange,” considering both weightings. These menus stand out for achieving an adequate balance between all the evaluated sustainability dimensions, ranking among the top 10% of the best-rated menus in terms of political and economic dimensions. Notably, according to this ranking, the ten most sustainable menus are free of animal-based ingredients, reaffirming that vegetarian preparations tend to be associated with lower costs and have a lesser impact on CF and WF, showing a trend toward local and vegetarian ingredients. In contrast, the menu classified as the least sustainable according to this ranking, for both weightings, is n°54: “Cabbage with mixed lettuces,” “Roast beef with mashed potatoes,” and “Baked milk.” This menu stands out for its high proportion of animal-based ingredients and for significantly impacting the environmental dimension. Its classification as the second worst according to the NRF 9.3 index is also relevant, negatively affecting the social dimension of the evaluation.

The evaluation of the menus in the indicators rankings reveals that, under both weightings (*s*_1_ and *s*_2_), menus n°4, n°53, and n°10 stand out for their sustainability. For example, menu n°4, which includes “Lettuce with carrot,” “Noodles with sauce and vegetables,” and “Apple,” presents one of the lowest water footprints and waste generation among the analyzed menus. Despite containing animal-based ingredients, this menu is characterized by a lower presence of such ingredients. Similarly, menu n°10, which consists of “Cabbage with mixed lettuces,” “Vegetarian potato pie,” and “Orange,” ranks among the menus with the least waste generation and the greatest use of local ingredients, thus positioning itself as a more sustainable menu. On the other hand, although menu n°53, composed of “Tomato with onion,” “Minestrone,” and “Apple,” does not register the lowest carbon and water footprints, its preference for local ingredients and contributions close to the average of each indicator also rank it among the most sustainable. Conversely, the menus considered the least sustainable, such as n°26, composed of “Tomato with lettuce,” “Fish with rice and vegetables,” and “Jelly,” and n°39, which includes “Tomato with green beans,” “Chicken with mashed potatoes and green beans,” and “Marbled jelly,” are characterized by high consumption of animal-based ingredients and a significant water footprint. Menus that incorporate animal-based ingredients, specifically fish, beef, or chicken, tend to occupy the lower positions in both rankings, mainly because their production is associated with considerable water consumption and greenhouse gas emissions, negatively impacting the evaluation of indicators.

The comprehensive evaluation of the menus, presented in Figure 2D, reveals that the ranking of dimensions shows more significant variability than the ranking of indicators. This is reflected in a standard deviation of 0.2540 for the dimensions compared to 0.2297 for the indicators. Additionally, the data distribution suggests symmetry around the mean.

After individually analyzing the categories of preparations, it has been identified that the differences between the equitable and biased weightings (*s*_1_ and *s*_2_) are minimal in both rankings applied (*SDI*^1^ and *SDI*^2^), indicating that the weighting methods have a limited impact on the variability of the results for the case studied. These findings suggest that sustainability rankings are robust across different weighting approaches, reinforcing their reliability for decision-making in large-scale food services. However, a deeper exploration of these results is needed to understand their broader implications, particularly in terms of their applicability to sustainable food policies and their potential to influence menu design strategies.

## 5 Discussion

The findings indicate that the sustainability of a menu does not always align with the sustainability rankings of its individual components. This suggests that interactions between food items play a crucial role in determining overall sustainability. For instance, menu n°53: “Tomato with onion,” “Minestrone,” and “Apple,” was ranked among the most sustainable according to the indicators ranking, despite none of its individual dishes being highly ranked. This discrepancy highlights the need to evaluate sustainability at a holistic level, considering how different food components interact to balance nutritional, environmental, and economic impacts. This multidimensional perspective aligns with Gazan et al. (34), who showed that integrating diverse dimensions into unified food sustainability database enhaces the robustness of assessments.

A key aspect of the rankings is that lower variability makes it more difficult to distinguish which culinary preparations are more sustainable. When rankings show little dispersion, the differences between preparations become less perceptible, whereas greater variability allows for clearer distinctions. Although the variability observed with the TODIM method is not highly significant, a slight dispersion is present. Notably, rankings based on sustainability dimensions (*SDI*^1^) exhibited greater variability than those based on indicators, reinforcing the idea that evaluating entire menus rather than individual preparations provides a more comprehensive assessment of sustainability. Furthermore, the results confirm that the inclusion of a specific preparation does not automatically determine the sustainability level of the entire menu, emphasizing the importance of holistic evaluations.

**Figure 3:**
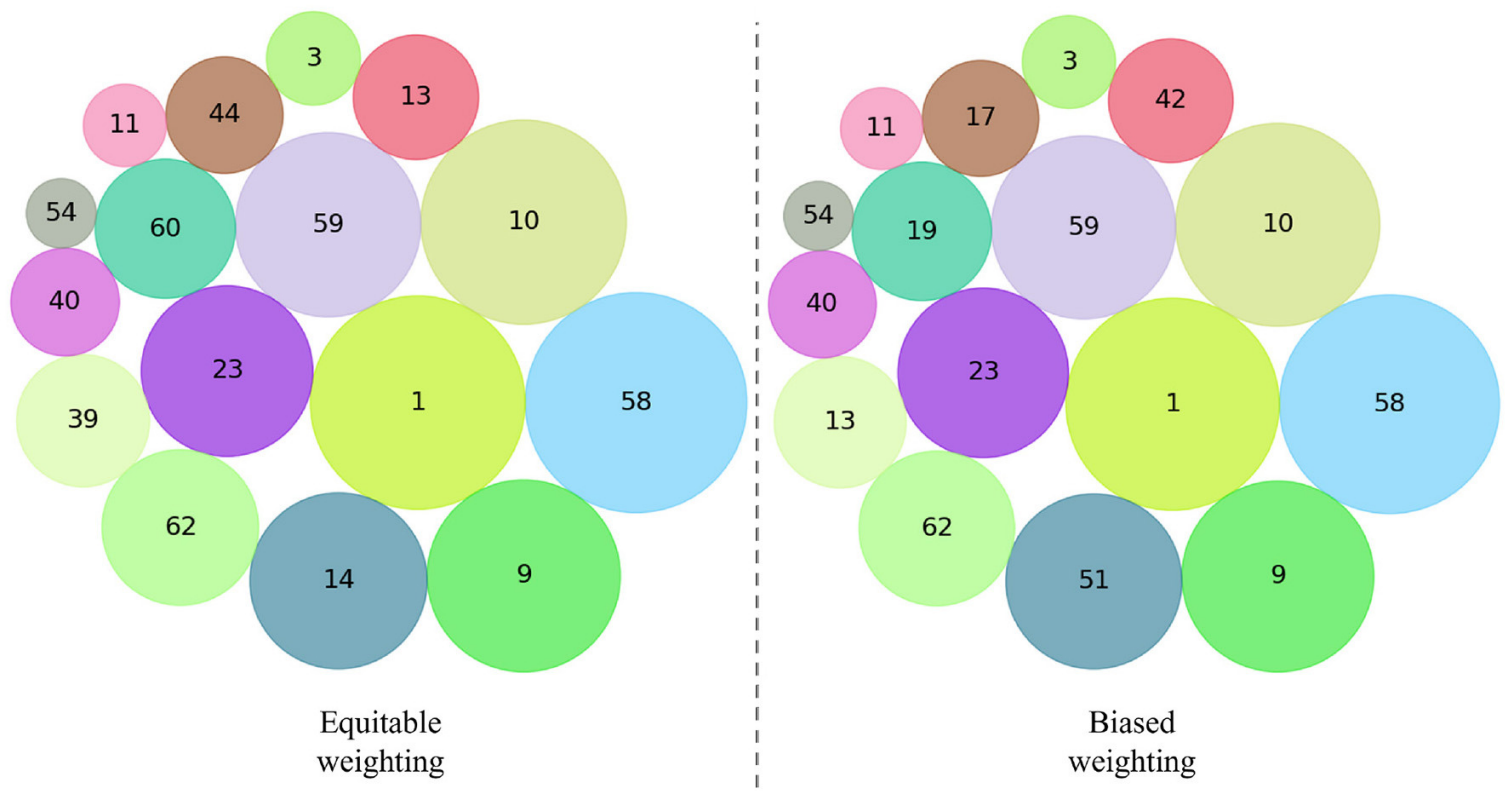
Bubble chart. Rankings of menus evaluated with sustainability dimensions.

Figure 3 represents a selection of the main menus from the sustainability dimensions ranking. Although only 16 menus are visualized in the figure, these correspond to the four most representative menus of each quartile of the ranking. It can be observed that the size of each circle indicates the menu's position within the ranking: the larger the size, the better the menu's classification in terms of sustainability. This graphical representation allows for a concise and visual appreciation of the menus leading in sustainability and those that, although included in the analysis, present greater opportunities for improvement. The strategic selection of menus from each quartile provides a clear understanding of the distribution of sustainability throughout the ranking.

Regarding the extremes of sustainability represented in the rankings, there is a notable consistency in the menus classified as the most and least sustainable between the first and last quartiles. The most sustainable menus remain constant despite the variation in weightings, with n°58, n°1, n°10, and n°9 leading the rankings. On the other hand, the menus categorized as the least sustainable are n°54, n°11, n°3, and n°40.

Among the least sustainable menus, menu n°11, which consists of “Beetroot with carrot and coriander,” “Roast beef with mixed vegetables,” and “Raspberry mousse,” as well as menu n°3, composed of “Mixed cabbage,” “Roast beef with chard cream,” and “Fruit jelly,” stand out for not meeting the minimum standards in the social dimension. According to the NRF 9.3 index, these menus offer virtually no nutritional value. They are also among those with the most negative impact on the environmental dimension and are classified within the 25% most costly menus of the study. Although menu n°11 might have a favorable ranking in the political dimension due to its use of local ingredients, this single advantage is insufficient to mitigate the negative effects in the other dimensions.

Additionally, menu n°40, composed of “Lettuce with tuna,” “Tomaticán with rice,” and “Strawberry,” also figures among the worst positioned in environmental terms, mainly due to its high impact on the “Species.yr” indicator. This menu is likewise among the most disadvantaged in the economic dimension. It is important to highlight that, according to the ranking, the least sustainable menus consistently involve the use of animal-based ingredients, which has a direct and considerable effect on the environmental impact of the preparations, regardless of the applied weighting.

The research reveals slight differences between the menus in the second and third quartiles (see Supplementary Table S7). For example, menu n°59, which includes “Beetroot with carrot,” “Roast chicken with spinach cream,” and “Mandarin,” contains animal-based ingredients. This detail suggests a slightly higher environmental impact than those based on legumes; nonetheless, menu n°59 falls within an average range compared to the other evaluated menus. In the economic dimension, this menu shows a lower acquisition cost than the average, but not low enough to classify it among the most economical. In terms of nutritional quality, while it offers a positive contribution, it does not particularly stand out, unlike the menus in the first quartile. For the technological dimension, the situation is similar; the menu does not show any particularities that make it stand out. However, in the political dimension, this menu achieves a significantly positive impact due to the extensive use of local ingredients, allowing it to partially offset the negative repercussions in the other dimensions.

Similarly, menu n°13, composed of “Cabbage with beetroot,” “Beef stew with noodles,” and “Fruit jelly,” despite being classified as one of the best in the social dimension and remaining accessible within the economic dimension, faces significant challenges in the environmental dimension. Specifically, its carbon footprint is considerable, mainly due to the use of animal-based ingredients in the main course and, in the dessert case, a more intensive preparation that implies less efficient use of ingredients. Although this menu does not occupy a prominent position in the overall ranking, it exemplifies how the different dimensions of sustainability interrelate and contribute to each other, creating a balanced menu that meets the minimum standards of each evaluated dimension.

While the methodology proposed in this study was developed and validated within the Chilean context, its generalizability to other cultural and geographical settings warrants further consideration. As observed in the Chinese case study by Li et al. (33), sustainability indicators must often be adapted to reflect regional dietary habits, ingredient availability, and socio-political priorities. Additionally, the relative weighting of dimensions may vary significantly across countries or institutions, suggesting the need for contextual calibration.

Moreover, despite the comprehensiveness of the index, certain limitations persist. As noted by Gazan et al. (34), compiling accurate and harmonized data across nutritional, economic, environmental, and social metrics is a major challenge. In many cases, data gaps, particularly in cultural acceptability, contaminants, or preparation techniques, may affect the completeness of assessments. Therefore, future applications of this index should account for local data constraints and consider stakeholder input to ensure relevance and robustness.

The discussion highlights the importance of a comprehensive sustainability assessment, covering environmental impact as well as nutritional, social, economic, political, and technological aspects. The results of this study indicate that the adoption of sustainable food practices transcends the environmental dimension, playing a crucial role in promoting health and overall wellbeing. The choice of ingredients, the combination of menu dishes, and culinary practices exemplify how informed decisions can foster a positive impact beyond the kitchen.

Therefore, food systems must recognize and adopt these sustainable practices, not as an isolated facet but as a global approach essential in food production and consumption. The results demonstrated that implementing sustainable food practices should not be limited to the environmental dimension but also enhance their benefits to health, economic stability, and social wellbeing.

## 6 Conclusions

Sustainability is key to mitigating the environmental impact of the food chain, a challenge amplified by the climate crisis. This study recognizes the need for a multidimensional analysis of sustainability, encompassing various indicators for the sustainability dimensions and designing a comprehensive index to evaluate diet sustainability through culinary preparations. This index can serve as a practical tool for institutions such as school or university canteens, public procurement agencies, or menu planners in food services to assess and improve the sustainability of their offerings in a systematic and informed manner. Its implementation may support actions such as menu reformulation, ingredient substitution, procurement planning, or sustainability reporting in institutional food services.

Throughout this research, different sustainability dimensions and indicators were evaluated. It was highlighted that sustainability is not limited to environmental aspects such as carbon and water footprints; it transcends to include the origin of ingredients, waste management, nutritional quality, and efficiency in food consumption. This represents an opportunity to promote health and wellbeing through responsible and conscious culinary decisions.

The analysis in this research showed a comparison between weightings established by experts and equitable weightings, evidencing reduced variability, thus allowing uniform weightings to serve as a reliable reference point for future evaluations. The results indicated that preparations with a high presence of animal products, especially meat, are less sustainable, emphasizing the impact of ingredient origin and transportation. It was evidenced that environmental aspects are only a part of sustainability, demonstrating the need to incorporate various aspects in the study to encompass the impact of food fully. Food systems must value and integrate these practices to facilitate an effective transition toward dietary patterns that harmoniously balance all dimensions of sustainability for a more resilient future. Furthermore, the index may be adapted by practitioners to reflect local priorities or institutional goals, increasing its relevance and applicability in diverse contexts. In conclusion, this study highlights the multidimensionality of sustainability and contributes to developing more sustainable food practices.

For the development of future work, it would be beneficial to explore a broader range of culinary preparations and menus, with a special focus on increasing vegetarian and vegan options, to assess their comparative impact on diet sustainability. Additionally, it would be advantageous to incorporate new indicators that measure the impact of food practices. Among the indicators to consider are land use, which measures the surface area required to produce food; energy consumption, which evaluates the total energy consumed in food production and preparation; access to nutritious foods; food security; the adoption of sustainable technologies in food production; social acceptance; and preparation time, among others. Moreover, strengthening the set of technical indicators by exploring innovations in food preparation, processing, or service could improve the index's ability to identify opportunities for optimization and to support actionable improvements in sustainable diet practices. For instance, evaluating the impact of low-energy cooking techniques could provide relevant insights for strengthening the technical dimension. Including these indicators will not only enrich the analysis of diet sustainability but also facilitate the formulation of more effective and specific strategies to promote sustainable food practices at the national level. Considering the seasonality of ingredient availability may also help refine the index and improve its applicability across different contexts. Overall, this work reinforces the value of integrative and flexible evaluation tools to support the transition toward more sustainable and resilient food systems.

## Data Availability

The original contributions presented in the study are included in the article/Supplementary material, further inquiries can be directed to the corresponding author.

## References

[1] Duchin F. Sustainable consumption of food: a framework for analyzing scenarios about changes in diets. J Ind Ecol. (2005) 9:99–114. 10.1162/108819805408470740187085

[2] Vermeulen SJ Campbell BM Ingram JSI. Climate change and food systems. Annu Rev Environ Resour. (2012) 37:195–222. 10.1146/annurev-environ-020411-130608

[3] Burlingame B Dernini S. Sustainable diets and biodiversity: directions and solutions for policy. Res Action. (2012) 309. Available online at: https://www.fao.org/4/i3004e/i3004e.pdf

[4] Springmann M Godfray HCJ Rayner M Scarborough P. Analysis and valuation of the health and climate change cobenefits of dietary change. Proc Nat Acad Sci. (2016) 113:4146–51. 10.1073/pnas.1523119113 27001851 PMC4839446

[5] Sokolow J Kennedy G Attwood S. Managing Crop tradeoffs: a methodology for comparing the water footprint and nutrient density of crops for food system sustainability. J Clean Prod. (2019) 225:913–27. 10.1016/j.jclepro.2019.03.056

[6] Vanham D. Water resources for sustainable healthy diets: state of the art and outlook. Water. (2020) 12:3224. 10.3390/w12113224

[7] Esteve-Llorens X Darriba C Moreira MT Feijoo G González-García S. Towards an environmentally sustainable and healthy Atlantic dietary pattern: life cycle carbon footprint and nutritional quality. Sci Total Environ. (2019) 646:704–15. 10.1016/j.scitotenv.2018.07.264 30059930

[8] FAO. Definition of sustainable diets. International Scientific Symposium. Biodiversity and sustainable diets united against hunger. Rome, Italy: FAO Headquarters (2010).

[9] Cencic A Chingwaru W. The role of functional foods, nutraceuticals, and food supplements in intestinal health. Nutrients. (2010) 2:611–25. 10.3390/nu2060611 22254045 PMC3257668

[10] Meybeck A Gitz V. Sustainable diets within sustainable food systems. Proc Nutr Soc. (2017) 76:1–11. 10.1017/S0029665116000653 28195528

[11] Bautista S Narvaez P Camargo M Chery O Morel L. Biodiesel-TBL+: a new hierarchical sustainability assessment framework of PC&I for biodiesel production – Part I. Ecol Indic. (2016) 60:84–107. 10.1016/j.ecolind.2015.06.02033317507

[12] Herrero M Thornton PK Mason-D'Croz D Palmer J Benton TG Bodirsky BL. Innovation can accelerate the transition towards a sustainable food system. Nat Food. (2020) 1:266–72. 10.1038/s43016-020-0074-1

[13] El Bilali H. Research on agro-food sustainability transitions: where are food security and nutrition? Food Secur. (2019) 11:559–77. 10.1007/s12571-019-00922-1

[14] Nemecek T Jungbluth N i Canals LM Schenck R. Environmental impacts of food consumption and nutrition: where are we and what is next? Int J Life Cycle Assess. (2016) 21:607–20. 10.1007/s11367-016-1071-3

[15] Sturtewagen L De Soete W Dewulf J Lachat C Lauryssen S Heirman B. Resource use profile and nutritional value assessment of a typical Belgian meal, catered or home cooked, with pork or Quorn^®^ as protein source. J Clean Prod. (2016) 112:196–204. 10.1016/j.jclepro.2015.09.006

[16] Wickramasinghe K Rayner M Goldacre M Townsend N Scarborough P. Environmental and nutrition impact of achieving new School Food Plan recommendations in the primary school meals sector in England. BMJ Open. (2017) 7:e013840. 10.1136/bmjopen-2016-013840 28381419 PMC5691295

[17] Hoefkens C Verbeke W Aertsens J Mondelaers K Van Camp J. The nutritional and toxicological value of organic vegetables: consumer perception versus scientific evidence. Br Food J. (2009) 111:1062–77. 10.1108/00070700920992916

[18] Hyska J Burazeri G Menza V Dupouy E. Assessing nutritional status and nutrition-related knowledge, attitudes and practices of Albanian school children to support school food and nutrition policies and programmes. Food Policy. (2020) 96:101888. 10.1016/j.foodpol.2020.101888

[19] Sullivan VS Smeltzer ME Cox GR MacKenzie-Shalders KL. Consumer expectation and responses to environmental sustainability initiatives and their impact in foodservice operations: a systematic review. J Hum Nutr Dietet. (2021) 34:994–1013. 10.1111/jhn.12897 34050994

[20] Soler C Moré N. Menú 2030. Transformar el menú para transformar el sistema alimentario. (2020).

[21] Engelmann T Speck M Rohn H Bienge K Langen N Howell E. Sustainability assessment of out-of-home meals: potentials and challenges of applying the indicator sets NAHGAST meal-basic and NAHGAST meal-pro. Sustainability. (2018) 10:562. 10.3390/su10020562

[22] Cambeses-Franco C González-García S Calvo-Malvar M Benítez-Estévez AJ Leis R Sánchez-Castro J. A clustering approach to analyse the environmental and energetic impacts of Atlantic recipes - a Galician gastronomy case study. J Clean Prod. (2023) 383:135360. 10.1016/j.jclepro.2022.135360

[23] Martinez S Alvarez S Martinez Marin R Delgado MdM. Feeding children with environmentally based dietary guidelines: the Nitrogen Footprint of school lunch menus adhering to the Spanish dietary guidelines. Sci Total Environ. (2022) 848:157796. 10.1016/j.scitotenv.2022.157796 35931147

[24] Gómez-Ramos A Rico Gonzalez M. The contribution of green public food procurement to sustainability: evidence from two case studies in Spain. Agroecol Sustain Food Syst. (2023) 47:1158–85. 10.1080/21683565.2023.2223555

[25] Stern AL Levine S Richardson SA Blackstone NT Economos C Griffin TS. Improving school lunch menus with multi-objective optimisation: nutrition, cost, consumption and environmental impacts. Public Health Nutr. (2023) 26:1715–27. 10.1017/S1368980023000927 37165566 PMC10410403

[26] Ernstoff A Tu Q Faist M Del Duce A Mandlebaum S Dettling J. Comparing the environmental impacts of meatless and meat-containing meals in the United States. Sustainability. (2019) 11:6235. 10.3390/su11226235

[27] Castañé S Antón A. Assessment of the nutritional quality and environmental impact of two food diets: a Mediterranean and a vegan diet. J Clean Prod. (2017) 167:929–37. 10.1016/j.jclepro.2017.04.121

[28] Benedetti I Laureti T Secondi L. Choosing a healthy and sustainable diet: a three-level approach for understanding the drivers of the Italians' dietary regime over time. Appetite. (2018) 123:357–66. 10.1016/j.appet.2018.01.004 29330002

[29] Fulgoni VL Keast DR Drewnowski A. Development and validation of the nutrient-rich foods index: a tool to measure nutritional quality of foods. J Nutr. (2009) 139:1549–54. 10.3945/jn.108.101360 19549759

[30] Guenther PM Casavale KO Reedy J Kirkpatrick SI Hiza HAB Kuczynski KJ. Update of the healthy eating index: HEI-2010. J Acad Nutr Diet. (2013) 113:569–80. 10.1016/j.jand.2012.12.016 23415502 PMC3810369

[31] Tepper S Geva D Shahar DR Shepon A Mendelsohn O Golan M. The SHED index: a tool for assessing a sustainable HEalthy diet. Eur J Nutr. (2021) 60:3897–909. 10.1007/s00394-021-02554-8 33904997

[32] Luzzani G. The sustainability of diets: current understanding and shortcomings. Curr Opin Environ Sci Health. (2022) 30:100398. 10.1016/j.coesh.2022.100398

[33] Li Y Filimonau V Wang L Cheng S. A set of preliminary indicators for holistic sustainability assessment of household food consumption in rural and urban China. Resour Conserv Recycl. (2023) 188:106727. 10.1016/j.resconrec.2022.106727

[34] Gazan R Barré T Perignon M Maillot M Darmon N Vieux F. A methodology to compile food metrics related to diet sustainability into a single food database: application to the French case. Food Chem. (2018) 238:125–33. 10.1016/j.foodchem.2016.11.083 28867082

[35] Hatjiathanassiadou M Rolim PM Seabra LMJ. Nutrition and its footprints: Using environmental indicators to assess the nexus between sustainability and food. Front Sustain Food Syst. (2023) 6:1078997. 10.3389/fsufs.2022.1078997

[36] Cáceres RP Troncoso PC Buhring BR Lataste QC. Sustainable food dishes: selection of indicators for their evaluation and communication in Chilean foodservices. Int J Gastron Food Sci. (2024) 35:100873. 10.1016/j.ijgfs.2024.100873

[37] Cáceres-Rodríguez P Jara M Parra-Soto S Troncoso-Pantoja C Lataste-Quintana C. Preparaciones culinarias ¿cómo determinar cuándo forman parte de una dieta sustentable? Rev chilena nutr. (2023) 50:86–97. 10.4067/S0717-7518202300010008627315006

[38] García-Leal J Espinoza Pérez AT Vásquez OC. Towards the sustainable massive food services: an optimization approach. Soc-Econ Plann Sci. (2023) 87:101554. 10.1016/j.seps.2023.101554

[39] Huijbregts MAJ Steinmann ZJN Elshout PMF Stam G Verones F Vieira M. ReCiPe2016: a harmonised life cycle impact assessment method at midpoint and endpoint level. Int J Life Cycle Assess. (2016) 22:138–47. 10.1007/s11367-016-1246-y

[40] Drewnowski A. The Nutrient Rich Foods Index helps to identify healthy, affordable foods. Am J Clin Nutr. (2010) 91:1095S–101S. 10.3945/ajcn.2010.28450D 20181811

[41] Lee H Calvin K Dasgupta D Krinner G Mukherji A Thorne P. Climate change 2023: synthesis report. In: Contribution of working groups I, II and III to the sixth assessment report of the intergovernmental panel on climate change. The Australian National University (2023).

[42] Boulay AM Bare J Benini L Berger M Lathuilliére MJ Manzardo A. The WULCA consensus characterization model for water scarcity footprints: assessing impacts of water consumption based on available water remaining (AWARE). Int J Life Cycle Assess. (2018) 23:368–78. 10.1007/s11367-017-1333-831884286

[43] Batlle-Bayer L Bala A García-Herrero I Lemaire E Song G Aldaco R. The Spanish Dietary Guidelines: a potential tool to reduce greenhouse gas emissions of current dietary patterns. J Clean Prod. (2019) 213:588–98. 10.1016/j.jclepro.2018.12.215

[44] González-García S González-García R González Vázquez L Moreira MT Leis R. Tracking the environmental footprints of institutional restaurant service in nursery schools. Sci Total Environ. (2020) 728:138939. 10.1016/j.scitotenv.2020.138939 32361112

[45] Aldaya MM Ibañez FC Domínguez-Lacueva P Murillo-Arbizu MT Rubio-Varas M Soret B. Indicators and recommendations for assessing sustainable healthy diets. Foods. (2021) 10:999. 10.3390/foods10050999 34063236 PMC8147455

[46] Reid IR Bolland MJ. Calcium and/or vitamin D supplementation for the prevention of fragility fractures: who needs it? Nutrients. (2020) 12:1011. 10.3390/nu12041011 32272593 PMC7231370

[47] González-García S Esteve-Llorens X Moreira MT Feijoo G. Carbon footprint and nutritional quality of different human dietary choices. Sci Total Environ. (2018) 644:77–94. 10.1016/j.scitotenv.2018.06.339 29981520

[48] Schaubroeck T Ceuppens S Luong AD Benetto E De Meester S Lachat C. A pragmatic framework to score and inform about the environmental sustainability and nutritional profile of canteen meals, a case study on a university canteen. J Clean Prod. (2018) 187:672–86. 10.1016/j.jclepro.2018.03.265

[49] Garnett T. Where are the best opportunities for reducing greenhouse gas emissions in the food system (including the food chain)? Food Policy. (2011) 36:S23–32. 10.1016/j.foodpol.2010.10.010

[50] O'Neill C McCarthy MB O'Reilly S Alfnes F. Food interests, preferences and behaviours: a profile of the sustainable food consumer. Br Food J. (2023) 125:352–74. 10.1108/BFJ-09-2022-0762

[51] Friel S Dangour AD Garnett T Lock K Chalabi Z Roberts I. Public health benefits of strategies to reduce greenhouse-gas emissions: food and agriculture. Lancet. (2009) 374:2016–25. 10.1016/S0140-6736(09)61753-0 19942280

[52] Troncoso-Pantoja C Cáceres-Rodríguez P Amaya-Placencia A Lataste-Quintana C Valenzuela R. Exploring the meanings of food sustainability: an interpretive phenomenological analysis. Sustainability. (2023) 15:13548. 10.3390/su15181354834086917

[53] Cinelli M Kadziński M Miebs G Gonzalez M Słowiński R. Recommending multiple criteria decision analysis methods with a new taxonomy-based decision support system. Eur J Oper Res. (2022) 302:633–51. 10.1016/j.ejor.2022.01.011

[54] Gomes L Lima M. TODIMI basics and application to multicriteria ranking. Found Comput Decis Sci. (1991) 16:1–16.

[55] Gomes L Lima M. From modeling individual preferences to multicriteria ranking of discrete alternatives: a look at prospect theory and the additive difference model. Found Comput Decis Sci. (1992) 17:171–84.

[56] MacLean LC Ziemba WT. Handbook of the Fundamentals of Financial Decision Making. vol 4 Singapore: World Scientific (2013). 10.1142/9789814417358_others01

[57] Rangel LAD Gomes LFAM. Determinação do valor de referência do aluguel de imóveis residenciais empregando o Método TODIM. Pesquisa Operacional. (2007) 27:357–72. 10.1590/S0101-74382007000200009

[58] Bai C Kusi-Sarpong S Badri Ahmadi H Sarkis J. Social sustainable supplier evaluation and selection: a group decision-support approach. Int J Prod Res. (2019) 57:7046–67. 10.1080/00207543.2019.1574042

[59] Autran Monteiro Gomes LF González XI. Behavioral multi-criteria decision analysis: further elaborations on the Todim method. Found Comput Decis Sci. (2012) 37:3–8. 10.2478/v10209-011-0001-1

[60] Gomes LFAM Rangel LAD Maranhão FJC. Multicriteria analysis of natural gas destination in Brazil: an application of the TODIM method. Math Comput Model. (2009) 50:92–100. 10.1016/j.mcm.2009.02.013

[61] Saaty RW. The analytic hierarchy process–what it is and how it is used. Mathem Modell. (1987) 9:161–76. 10.1016/0270-0255(87)90473-8

[62] Organisation for Economic Co-operation and Development (OCDE). Obesity Update 2017. (2017). Available online at: https://www.oecd.org/els/health-systems/Obesity-Update-2017.pdf (Accessed February 10, 2024).

[63] PAHO. Ultra-processed food and drink products in Latin America: Trends, impact on obesity, policy implications (2015). Available online at: https://iris.paho.org/bitstream/handle/10665.2/7698/9789275318645_esp.pdf (Accessed January 15, 2025).

[64] Oficina de Estudios y Políticas Agrarias (ODEPA). Caracterización de la demanda de carne bovina y evaluación de bienes sustitutos (2007). Available online at: https://bibliotecadigital.odepa.gob.cl/handle/20.500.12650/3668 (Accessed January 5, 2025).

[65] Oficina de Estudios y Políticas Agrarias (ODEPA). Estrategia Nacional de Soberanía para la Seguridad Alimentaria. (2023). Available online at: https://soberaniaalimentaria.odepa.gob.cl/ (Accessed January 3, 2025).

[66] Villagrán MM. Boletín de hortalizas. Technical report, Oficina de Estudios y Políticas Agrarias (ODEPA). (2021).

[67] García Lizama ALE. Boletín de cereales - *Julio 2021*. Technical report, Oficina de Estudios y Políticas Agrarias (ODEPA. (2021).12265407

[68] Cáceres Rodríguez P Lataste Quintana C. Indicadores de transformación de alimentos: manual para su uso en el quehacer del nutricionista. (2021). Available online at: https://libros.uchile.cl/1252 (Accessed October 12, 2024).

[69] Oficina de Estudios y Políticas Agrarias (ODEPA). ODEPA Precios consumidor. Technical report, Ministerio de Agricultura. (2021).29668137

[70] ChileCompra. Mercado Público - *La plataforma de compras públicas y oportunidades de negocio del Estado de Chile* – *mercadopublico.cl* (2009). Available online at: https://www.mercadopublico.cl/Home (Accessed July 18, 2024).

[71] Jumbo. Supermercado | *Jumbo.cl* (2010). Available online at: https://www.jumbo.cl/ (Accessed March 23, 2024).

[72] SantaIsabel. Supermercado | *Santa Isabel.cl* (2011). Available online at: https://www.santaisabel.cl/ (Accessed March 23, 2024).

[73] Agricultural Research Service U. FoodData Central (2012). Available online at: https://fdc.nal.usda.gov/fdc-app.html (Accessed July 18, 2024).

[74] Durán Agüero S Onfray Centonzio P Correa Yáñez MJ Gamboa Menai S Cancino López V. Evaluación del nivel de participación del nutricionista en la prescripción dietética en hospitales públicos y privados de Chile. Perspect Nutr Humana. (2019) 21:71–79. 10.17533/udea.penh.v21n1a0639083843

[75] VRIIC VdIyc. Comité de Ética Institucional USACH - *cei.usach.cl* (2014). Available online at: https://www.cei.usach.cl/ (Accessed January 7, 2024).

[76] Kuzma S Bierkens MFP Lakshman S Luo T Saccoccia L Sutanudjaja EH. Aqueduct 4.0: Updated Decision-Relevant Global Water Risk Indicators. World Resources Institute (2023). 10.46830/writn.23.00061

[77] Cáceres P Strasburg VJ Morales M Huentel C Jara C Solís Y. Determination of eco-efficiency in food waste generated at the household level: pilot case in Chile. Rev Cienc Ambient. (2021) 55:295–310. 10.15359/rca.55-2.14

