# Supplementary material for: Sustainability diet index: a multi-criteria decision analysis proposal for culinary preparations—A case study
Source: Front Nutr. 2025 Jul 9;12:1586886. doi: 10.3389/fnut.2025.1586886 (PMC12283303; doi:10.3389/fnut.2025.1586886)
Supplement: Supplementary file 1 [file Data_Sheet_1.pdf]

Supplementary Material

1 INDICATOR EVALUATION DETAILS

Table S1. Evaluation of Indicators - Starters. CF: carbon footprint, WF: water footprint.

N°	Starter	CF (CO2 eq)	WF (m3)	Use of local ingredients (%)	Presence of animal foods (%)	Red meat presence (%)	Waste (%)
1	Beetroot with onion and carrot	0.086	0.437	89.266	0	0	15.481
2	Red cabbage with peppers	0.167	0.289	4.520	0	0	13.994
3	Celery with avocado and other vegetables	0.301	0.627	2.111	0	0	14.624
4	Lettuce with broad beans	0.154	0.243	26.728	0	0	19.977
5	Cream of vegetable soup	0.096	0.179	67.010	18.557	0	17.287
6	Tomato with coriander	0.264	0.221	1.242	0	0	54.736
7	Beef consomme	1.565	0.390	16.867	69.880	60.241	4.401
8	Cabbage mix	0.115	0.145	4.938	0	0	13.889
9	Lettuce with corn	0.126	0.230	19.792	0	0	15.547
10	Beetroot with coriander	0.056	0.333	96.981	0	0	14.663
11	Spinach with carrot	0.043	0.112	95.652	0	0	42.522
12	Tomato with green bean	0.424	0.122	31.579	0	0	55.301
13	Broccoli with cauliflower	0.099	0.178	96.970	0	0	0.000
14	Lettuce with spinach	0.117	0.186	19.792	0	0	16.766
15	Poultry consomme	0.167	0.397	10.625	63.125	0	18.773
16	Tomato with cucumber	0.428	0.249	23.529	0	0	47.836
17	Lettuce with carrot	0.117	0.225	15.217	0	0	10.728
18	Celery with olives	0.128	0.155	5.714	0	0	20.245
19	Lettuce with spring salad	0.107	0.179	5.793	0	0	9.535
20	Beetroot with carrot	0.069	0.364	97.531	0	0	16.381
21	Green beans with corn	0.185	0.137	96.610	0	0	47.272
22	Lettuce with bell pepper mix	0.200	0.438	4.167	0	0	11.359
23	Celery, spinach and olives	0.140	0.163	22.353	0	0	26.784
24	Lettuce with mushroom	0.119	0.256	4.396	0	0	8.242
25	Meat pie	1.595	0.889	1.340	42.753	40.601	0.713
26	Beetroot with onion	0.064	0.340	90.141	0	0	14.290
27	Celery with spinach	0.133	0.146	23.457	0	0	26.856
28	Cabbage with beetroot	0.137	0.274	30.631	0	0	14.511
29	Cabbage mix with corn	0.140	0.208	22.222	0	0	20.364
30	Celery with carrot	0.130	0.160	18.421	0	0	20.432
31	Noodle soup	0.267	0.140	11.236	0	0	44.234
32	Tomato with lettuce	0.229	0.233	3.376	0	0	24.921
33	Carrot with cucumber	0.360	0.197	96.970	0	0	21.515
34	Lettuce with green beans and carrot	0.184	0.165	26.316	0	0	18.527
35	Cream of pumpkin soup	0.095	0.326	0.000	17	0	31.507
36	Celery with carrot and olive	0.137	0.177	17.500	0	0	20.677
37	Lettuce with spinach and carrot	0.126	0.208	27.358	0	0	17.420
38	Lettuce with tuna	0.453	0.209	9.929	35.461	0	7.000
39	Vegetarian pizza	0.283	0.921	22.930	5.096	0	12.964
40	Pizza	0.398	1.040	21.557	11.976	0	13.616
41	Artichokes	0.210	0.091	3.053	0	0	62.309
42	Spinach with celery and corn	0.196	0.183	37.681	0	0	30.583
43	Cabbage mix with lettuce	0.167	0.431	52.406	0	0	11.230
44	Beetroot with carrot and coriander	0.083	0.207	91.346	0	0	14.041
45	Lettuce with carrot and peas	0.129	0.339	75.127	0	0	11.497
46	Carrot with spinach and spring	0.129	0.308	80.203	0	0	30.838
47	Tomato with onion	0.300	0.160	4.520	0	0	50.276
48	Celery with corn	0.211	0.191	23.188	0	0	25.457
49	Celery with cabbage	0.221	0.217	11.814	0	0	16.167
50	Cabbage mix with olives	0.187	0.177	3.865	0	0	15.265
51	Cabbage with corn and celery	0.209	0.209	25.551	0	0	23.918
52	Lettuce with celery and carrot	0.188	0.179	17.512	0	0	15.509
53	Cabbage with olives	0.176	0.192	4.061	0	0	15.803
54	Lettuce with carrot and corn	0.154	0.235	49.746	0	0	21.944
55	Celery with peppers	0.310	0.468	3.865	0	0	20.278
56	Spinach, celery with corn and olives	0.134	0.147	60.793	0	0	36.615

Table S2. Evaluation of Indicators - Main courses. CF: carbon footprint, WF: water footprint.

N°	Main courses	CF (CO2 eq)	WF (m3)	Use of local ingredients (%)	Presence of animal foods (%)	Red meat presence (%)	Waste (%)
1	Roasted turkey with creamed spinach	1.249	1.006	23.227	67.506	0	22.951
2	Beef stew with rice	4.775	2.880	3.337	37.083	37.083	7.294
3	Charquican	4.699	1.336	7.969	32.751	32.751	15.353
4	Chicken stewed with quinoa	0.669	2.266	3.509	57.416	0	18.236
5	Beef stew with noodles	4.692	1.003	28.229	55.351	55.351	4.794
6	Chicken casserole	0.660	1.846	4.314	47.059	0	19.426
7	Chilean bean soup with spaghetti	0.251	0.359	23.569	0	0	33.049
8	Vegetable pudding with boiled potatoes	0.500	0.467	56.121	12.121	0	17.548
9	Noodles with seafood sauce	0.421	0.312	30.056	61.224	0	43.815
10	Stewed chickpeas	0.231	1.685	3.191	0	0	17.092
11	Meat and vegetable stew	2.904	1.235	7.101	26.627	26.627	17.227
12	Pork stew with quinoa	1.258	2.264	4.545	49.587	0	21.392
13	Stroganoff with rice	3.150	3.487	3.267	41.742	32.668	1.911
14	Baked fish with mashed potatoes	1.291	0.591	0.212	37.500	0	11.705
15	Stewed lentils	0.207	0.570	3.368	0	0	16.755
16	Chicken and green peas stew with mashed potatoes	0.701	4.469	2.149	49.587	0	12.887
17	Chicken stew with rice	0.732	4.612	3.328	45.386	0	16.190
18	Valencian rice	0.623	3.912	9.381	46.018	0	26.976
19	Pantrucas	3.752	1.028	7.394	47.535	42.254	7.812
20	Ajiaco	5.560	1.268	5.907	53.446	50.633	6.904
21	Chicken and green peas stew with rice	0.633	3.066	2.058	51.458	0	16.545
22	Beef tenderloin with corn pudding	7.527	4.048	64.475	64.960	13.841	14.278
23	Roasted meat with creamed spinach	4.688	1.083	33.890	58.431	50.083	19.511
24	Oven-roasted ribs with vegetable medley	1.943	2.144	7.905	50.188	0	21.984
25	Chicken stew with rice	0.555	2.686	2.105	42.105	0	14.856
26	Beef casserole	5.515	1.800	4.741	51.724	51.724	7.448
27	Corn pie	19.927	9.102	53.603	56.830	17.928	24.862
28	Mixed grill with chilean salad	4.298	2.383	13.224	53.878	16.327	23.406
29	Potato pie	2.442	0.790	6.552	33.385	21.841	9.886
30	Beans with mazamorra	0.490	0.575	40.564	24.515	0	35.877
31	Seafood soup	0.782	0.255	1.985	54.962	0	26.094
32	Beef with coriander and vegetable medley	5.521	1.283	16.706	52.399	52.399	4.253
33	Humitas x2	0.508	1.056	87.364	6.043	0	43.767
34	Roast chicken with rice	0.691	4.431	3.642	49.669	0	13.211
35	Oven-baked fish with spring quinoa	1.041	0.880	5.176	55.453	0	9.239
36	Noodles with spring sauce	2.371	0.522	60.571	26.667	26.667	5.816
37	Pork stew with au gratin potatoes	1.427	1.637	3.511	42.748	0	20.841
38	Chicken stew with spring rice	0.741	4.622	5.433	44.053	0	16.651
39	Zucchini squash stew with roasted potatoes	4.724	1.300	53.113	43.709	39.735	26.234
40	Chicken stew with quinoa	2.999	0.825	36.595	30.831	24	7.316
41	Noodles with bontux sauce	0.575	1.818	4.457	58.140	0	19.760
42	Tomatican with rice	0.497	0.917	32.618	55.794	0.000	13.445
43	Minestrone	4.947	4.666	7.632	39	39	9.161
44	Roasted chicken with mixed mash	0.284	0.384	31.541	0.000	0	25.544
45	Beef stew with whole rice	0.534	1.219	2.415	42.614	0.000	18.869
46	Pork stew with rice	4.690	1.684	3.661	49.917	50	4.862
47	Beef roast with creamed corn	1.391	4.786	5.175	40.067	0.000	16.923
48	Pork stew with au gratin potatoes	1.393	1.634	3.577	43.546	0	21.240
49	Pea and pork cutlet stew with curry rice	1.384	4.685	2.559	43.876	0	13.554
50	Chicken and green peas stew with rice noodles	0.557	1.068	30.297	59.406	0	15.439
51	Beef fajitas x2	3.941	1.777	8.343	27.057	27.057	8.906
52	Beef stew with mixed mash	4.765	1.513	1.421	36.066	32.787	17.641
53	Oven-roasted turkey with spring rice	0.852	3.855	4.576	50.847	0	9.826
54	Roasted chicken with quinoa	0.889	2.669	1.743	80.429	0	20.083
55	Roasted chicken with creamed spinach	0.973	2.161	22.581	72.303	0	29.298
56	Vegetable pudding with quinoa	0.294	1.157	55.319	13.298	0	13.363
57	Roasted turkey with vegetable medley	0.771	0.678	1.998	33.296	0	13.589
58	Zucchini squash stew with rice	1.997	3.937	56.928	17.321	11.547	3.304
59	Meat in mushroom sauce with noodles	5.645	1.289	29.647	59.908	55.300	0.132
60	Beans with pilco	0.177	0.284	22.673	0	0	42.742
61	Chicken lasagna with vegetables	0.872	1.675	35.795	50.000	0	17.333
62	Roasted meat with vegetable medley	5.547	1.309	21.102	48.387	48.387	6.452

Table S2. *(continued)*

N°	Main courses	CF (CO2 eq)	WF (m3)	Use of local ingredients (%)	Presence of animal foods (%)	Red meat presence (%)	Waste (%)
63	Poblano cream with breaded steak	4.004	1.792	4.329	43.290	25.974	3.923
64	Veracruz-style fish with vegetable rice	1.349	3.609	0	32.609	0	20.272
65	Mexican flautas	0.869	1.441	0	46.000	0	10.326
66	Sausage and bean stew	0.848	0.971	35.577	25.641	0	24.828
67	Ratatouille	0.784	1.628	29.848	0	0	23.112
68	Vegetable curry with rice	0.831	3.843	36.910	0	0	27.716
69	Vegetable lasagna	1.503	1.518	65.381	20.628	0	5.270
70	Quinoa risotto	0.623	2.299	32.203	8.475	0	1.944
71	Spinach risotto	0.366	4.445	24.632	0	0	14.838
72	Vegetarian corn pie	3.007	2.047	65.412	51.788	0	15.676
73	Roasted pulp with creamed chard	2.330	2.578	30.074	64.356	0	20.197
74	Vegetarian meat and vegetable stew	0.392	0.926	20.085	0	0	23.314
75	Vegetarian potato pie	0.459	0.394	17.259	11.844	0	13.821
76	Pork pulp with vegetable medley	2.261	2.772	17.546	58.896	0	21.993
77	Arab rice with goulash	4.761	3.090	14.869	43.732	43.732	3.014
78	Noodles with bolognese sauce	4.391	0.923	35.413	51.376	51.376	1.022
79	Roasted chicken with spring rice	0.906	4.566	5.036	57.554	0	15.717
80	Vegetarian tomatican with rice	0.575	3.303	24.643	0	0	16.527
81	Oven-baked pork pulp with mash	2.346	2.659	1.439	62.350	0	21.912
82	Oven-baked fish with rice	1.099	2.941	2.166	54.152	0	1.006
83	Vegetarian charquican	0.332	0.612	25.455	0	0	20.577
84	Stuffed zucchini with quinoa	4.435	2.042	40.521	33.175	33.175	3.289
85	Beef stew with mash	7.427	1.762	4.646	57.522	53.097	6.198
86	Vegetable pudding with rice	0.578	3.234	41.438	8.562	0	11.005
87	Swiss chard stew with roasted potatoes	0.391	0.527	27.829	2	0	14.102
88	Pork and green peas stew with noodles	2.377	2.591	26.082	56.140	0	18.129
89	Roasted chicken with vegetable medley	0.829	1.920	0.276	44.240	0	16.839
90	Noodles with mushroom and corn sauce	0.361	0.469	58.954	8.048	0	9.762
91	Vegetarian pantrucas	0.385	0.403	51.147	9.174	0	12.895
92	Beef with mushroom and au gratin potatoes	5.566	1.373	0.428	55.635	51.355	5.063

Table S3. Evaluation of Indicators - Desserts. CF: carbon footprint, WF: water footprint.

N°	Dessert	CF ($CO2$ eq)	WF (m3)	Use of local ingredients (%)	Presence of animal foods (%)	Red meat presence (%)	Waste (%)
1	Apple	0.041	-0.018	0	0	0	0
2	Semolina pudding	0.412	0.461	0	68.220	0	0
3	Orange	0.071	0.744	100	0	0	0
4	Fruit jelly	0.022	0.108	0	37.5	0	0
5	Pear	0.077	0.427	0	0	0	0
6	Pineapple	0.072	0.084	0	0	0	38.99
7	Brazo de reina	0.118	0.242	33.333	33.333	0	3.753
8	Roasted milk	0.106	0.073	40.541	67.568	0	4.565
9	Bavarian cream	0.035	0.079	0	100	0	0
10	Chocolate flan	0.749	0.325	33.146	83.708	0	3.100
11	Carrot cake	0.151	0.362	50	38	0	10.739
12	Vanilla flan	0.459	0.234	29.167	88.690	0	3.284
13	Caramel flan	0.459	0.234	29.167	88.690	0	3.284
14	Raspberry mousse	0.056	0.161	0	83.333	0	0
15	Pineapple jelly	0.015	0.052	0	37.5	0	24.369
16	Wheat with dried peaches	0.111	1.040	0	0	0	0
17	Marble jelly	0.075	0.109	0	100	0	0
18	Fried bananas with cream	0.723	2.547	0	15.556	0	0
19	Tricolor jellies	0.070	0.177	0	100	0	0
20	Candied pumpkin	0.033	0.210	0	0	0	41.6
21	Peach	0.075	0.491	0	0	0	0
22	Strawberry	0.095	0.956	0	0	0	0
23	Apricot	0.062	0.376	0	0	0	0
24	Cherry	0.114	4.631	0	0	0	0
25	Kiwi	0.104	0.350	0	0	0	18.35
26	Tangerine	0.117	0.904	0	0	0	0
27	Papaya	0.070	0.218	0	0	0	0
28	Banana	0.139	0.740	0	0	0	0
29	Mango	0.136	1.398	0	0	0	0
30	Raspberry	0.154	0.978	0	0	0	0
31	Fig	0.065	2.263	100	0	0	0
32	Melon	0.037	0.121	0	0	0	0
33	Watermelon	0.049	0.463	0	0	0	0

Table S4. Evaluation of Sustainability Dimensions - Starters. GWP: Global Warming Potential, NRF 9.3: Nutrient Rich Foods Index.

N°	Starter	Environmental			Economic		Social		Political (%)	Techno-logical
		GWP (CO_2 eq)	Water use (m3)	Species.yr	USD2013	Cost ($)	DALY	NRF 9.3		
1	Beetroot with onion and carrot	0.086	0.437	0.414	0.012	179.156	0.815	-0.097	89.266	6
2	Red cabbage with peppers	0.167	0.289	70.142	0.028	217.148	1.492	-0.107	4.520	5
3	Celery with avocado and vegetables	0.301	0.627	1.541	0.057	652.752	1.556	0.074	2.111	9
4	Lettuce with broad beans	0.154	0.243	0.721	0.025	383.197	0.951	-0.052	26.728	5
5	Cream of vegetable soup	0.096	0.179	0.531	0.014	231.986	0.415	-0.209	67.010	8
6	Tomato with coriander	0.264	0.221	0.433	0.073	287.478	0.745	-0.150	1.242	4
7	Beef consomme	1.565	0.390	37.310	0.066	389.697	2.145	0.126	16.867	9
8	Cabbage mix	0.115	0.145	0.456	0.021	138.351	0.703	0.173	4.938	4
9	Lettuce with corn	0.126	0.230	0.490	0.021	372.907	0.822	0.054	19.792	5
10	Beetroot with coriander	0.056	0.333	0.263	0.007	103.270	0.572	0.006	96.981	5
11	Spinach with carrot	0.043	0.112	0.130	0.004	152.506	0.389	0.036	95.652	5
12	Tomato with green bean	0.424	0.122	1.395	0.073	357.982	1.011	-0.070	31.579	5
13	Broccoli with cauliflower	0.099	0.178	0.205	0.014	140.788	0.653	0.052	96.970	5
14	Lettuce with spinach	0.117	0.186	0.470	0.020	280.567	0.818	0.040	19.792	5
15	Poultry consomme	0.054	0.109	34.992	0.008	81.747	0.523	-0.003	24.286	9
16	Tomato with cucumber	0.428	0.249	0.577	0.101	317.395	1.035	-0.056	23.529	5
17	Lettuce with carrot	0.117	0.225	0.498	0.021	259.353	0.801	-0.054	15.217	5
18	Celery with olives	0.128	0.155	0.453	0.025	335.129	0.649	-0.022	5.714	5
19	Lettuce with spring salad	0.107	0.179	0.458	0.019	231.899	0.704	-0.013	5.793	8
20	Beetroot with carrot	0.069	0.364	0.298	0.008	124.566	0.711	0.029	97.531	5
21	Green beans with corn	0.185	0.137	1.052	0.004	326.856	0.480	-0.005	96.610	5
22	Lettuce with bell pepper mix	0.200	0.438	209.273	0.029	366.857	2.970	0.001	4.167	5
23	Celery, spinach and olives	0.140	0.163	0.477	0.026	388.439	0.770	0.033	22.353	6
24	Lettuce with mushroom	0.119	0.256	0.549	0.022	352.037	0.791	0.005	4.396	5
25	Meat pie	1.595	0.889	3.489	0.074	391.088	1.857	0.349	1.340	9
26	Beetroot with onion	0.064	0.340	0.307	0.009	113.476	0.648	0.013	90.141	5
27	Celery with spinach	0.133	0.146	0.438	0.025	361.679	0.728	0.048	23.457	5
28	Cabbage with beetroot	0.137	0.274	0.557	0.023	174.051	0.962	0.221	30.631	5
29	Cabbage mix with corn	0.140	0.208	0.508	0.023	313.131	0.853	0.257	22.222	5
30	Celery with carrot	0.130	0.160	0.439	0.025	322.929	0.700	0.007	18.421	5
31	Noodle soup	0.267	0.140	0.551	0.071	274.058	0.917	0.156	11.236	4
32	Tomato with lettuce	0.229	0.233	0.603	0.054	334.657	1.044	0.021	3.376	5
33	Carrot with cucumber	0.360	0.197	0.459	0.063	167.913	0.794	-0.031	96.970	5
34	Lettuce with green beans and carrot	0.184	0.165	0.927	0.020	297.528	0.893	-0.001	26.316	5
35	Cream of pumpkin soup	0.095	0.326	0.546	0.014	171.521	0.472	-0.098	0	6
36	Celery with carrot and olive	0.137	0.177	0.477	0.026	349.689	0.742	-0.007	17.500	6
37	Lettuce with spinach and carrot	0.126	0.208	0.494	0.021	295.127	0.911	0.055	27.358	6
38	Lettuce with tuna	0.453	0.209	0.883	0.124	917.111	1.118	0.508	9.929	6
39	Vegetarian pizza	0.283	0.921	1.641	0.047	562.128	0.740	0.205	22.930	7
40	Pizza	0.398	1.040	2.054	0.064	643.678	0.830	0.177	21.557	8
41	Artichokes	0.210	0.091	0.996	0.029	390.493	1.637	0.608	3.053	4
42	Spinach with celery and corn	0.196	0.183	0.671	0.032	561.412	0.931	0.008	37.681	6
43	Cabbage mix with lettuce	0.167	0.431	0.619	0.024	211.067	0.930	-0.038	52.406	5
44	Beetroot with carrot and coriander	0.083	0.207	0.347	0.010	105.134	0.451	-0.171	91.346	6
45	Lettuce with carrot and peas	0.129	0.339	0.816	0.020	274.016	0.871	-0.053	75.127	6
46	Carrot with spinach and spring	0.129	0.308	0.551	0.017	287.644	0.879	-0.028	80.203	6
47	Tomato with onion	0.300	0.160	0.591	0.077	262.409	0.861	-0.143	4.520	5
48	Celery with corn	0.211	0.191	0.736	0.037	577.363	0.949	-0.027	23.188	5
49	Celery with cabbage	0.221	0.217	0.825	0.040	392.742	1.045	0.000	11.814	6
50	Cabbage mix with olives	0.187	0.177	0.804	0.031	245.195	0.943	-0.079	3.865	6
51	Cabbage with corn and celery	0.209	0.209	0.755	0.035	521.385	1.014	0.089	25.551	6
52	Lettuce with celery and carrot	0.188	0.179	0.738	0.033	360.953	0.985	-0.101	17.512	6
53	Cabbage with olives	0.176	0.192	0.795	0.029	270.549	0.906	-0.051	4.061	5
54	Lettuce with carrot and corn	0.154	0.235	0.586	0.022	426.287	0.871	-0.031	49.746	6
55	Celery with peppers	0.310	0.468	279.114	0.047	569.296	3.813	-0.095	3.865	5
56	Spinach, celery with corn and olives	0.134	0.147	0.474	0.021	475.644	0.585	0.066	60.793	7

Table S5. Evaluation of Sustainability Dimensions - Main courses. GWP: Global Warming Potential, NRF 9.3: Nutrient Rich Foods Index.

N°	Main courses	Environmental			Economic		Social		Political (%)	Techno-logical
		GWP (CO2 eq)	Water use (m3)	Species.yr	USD2013	Cost ($)	DALY	NRF 9.3		
1	Roasted turkey with creamed spinach	1.249	1.006	41.105	0.210	1972.019	2.094	0.391	23.227	11
2	Beef stew with rice	4.775	2.880	77.797	0.210	1232.911	6.328	0.599	3.337	13
3	Charquican	4.699	1.336	8.407	0.206	1281.094	5.942	0.572	7.969	14
4	Chicken stewed with quinoa	0.669	2.266	37.892	0.114	1033.753	1.401	0.357	3.509	13
5	Beef stew with noodles	4.692	1.003	77.494	0.218	1174.533	6.205	0.614	28.229	12
6	Chicken casserole	0.660	1.846	37.744	0.095	786.812	1.512	0.252	4.314	13
7	Chilean bean soup with spaghetti	0.251	0.359	70.815	0.042	561.353	1.635	-0.079	23.569	13
8	Vegetable pudding with boiled potatoes	0.500	0.467	71.112	0.055	813.095	2.388	0.002	56.121	12
9	Noodles with seafood sauce	0.421	0.312	37.215	0.103	1092.671	1.433	0.391	30.056	13
10	Stewed chickpeas	0.231	1.685	162.471	0.031	438.348	2.247	0.232	3.191	12
11	Meat and vegetable stew	2.904	1.235	40.151	0.133	879.624	4.172	0.273	7.101	16
12	Pork stew with quinoa	1.258	2.264	142.750	0.195	1301.195	2.939	-0.057	4.545	10
13	Stroganoff with rice	3.150	3.487	144.980	0.157	946.676	4.957	0.111	3.267	16
14	Baked fish with mashed potatoes	1.291	0.591	3.972	0.259	1132.127	1.603	0.549	0.212	12
15	Stewed lentils	0.207	0.570	36.020	0.027	433.311	0.997	0.378	3.368	12
16	Chicken and green peas stew with mashed potatoes	0.701	4.469	37.156	0.086	661.882	1.116	0.218	2.149	11
17	Chicken stew with rice	0.732	4.612	141.309	0.090	701.144	2.368	0.481	3.328	10
18	Valencian rice	0.623	3.912	71.526	0.086	867.165	1.558	0.381	9.381	13
19	Pantrucas	3.752	1.028	41.119	0.160	936.730	4.779	0.643	7.394	11
20	Ajiaco	5.560	1.268	44.105	0.234	1300.300	6.798	0.596	5.907	11
21	Chicken and green peas stew with rice	0.633	3.066	37.193	0.087	652.542	1.160	0.204	2.058	11
22	Beef tenderloin with corn pudding	5.796	1.973	14.350	0.455	5322.081	9.503	-3.251	64.475	9
23	Roasted meat with creamed spinach	4.688	1.083	42.928	0.198	1174.276	5.810	0.388	33.890	12
24	Oven-roasted ribs with vegetable medley	1.943	2.144	6.055	0.286	1677.589	2.383	-0.629	7.905	15
25	Chicken stew with rice	0.555	2.686	71.709	0.074	596.003	1.541	0.139	2.105	12
26	Beef casserole	5.515	1.800	43.908	0.224	1253.160	6.677	0.880	4.741	12
27	Corn pie	5.486	2.395	14.140	1.072	11572.585	26.208	4.287	53.603	10
28	Mixed grill with Chilean salad	4.298	2.383	8.884	0.321	1700.752	5.477	0.740	13.224	10
29	Potato pie	2.442	0.790	5.670	0.128	836.222	3.162	0.018	6.552	14
30	Beans with mazamorra	0.490	0.575	38.171	0.055	970.872	1.355	-0.209	40.564	13
31	Seafood soup	0.782	0.255	36.740	0.174	1248.025	1.407	0.236	1.985	12
32	Beef with coriander and vegetable medley	5.521	1.283	43.923	0.230	1345.554	6.875	0.610	16.706	17
33	Humitas x2	0.508	1.056	1.705	0.049	2658.010	2.605	1.128	87.364	5
34	Roast chicken with rice	0.691	4.431	2.314	0.085	622.989	0.822	0.201	3.642	9
35	Oven-baked fish with spring quinoa	1.041	0.880	71.567	0.227	1081.518	1.697	0.704	5.176	15
36	Noodles with spring sauce	2.371	0.522	4.664	0.148	873.725	3.288	0.689	60.571	14
37	Pork stew with au gratin potatoes	1.427	1.637	74.437	0.213	1240.379	2.514	-0.495	3.511	14
38	Chicken stew with spring rice	0.741	4.622	141.371	0.091	724.352	2.403	0.494	5.433	13
39	Zucchini squash stew with roasted potatoes	4.724	1.300	8.010	0.143	1045.197	4.397	0.225	36.595	13
40	Chicken stew with quinoa	2.999	0.825	40.114	0.095	846.098	1.614	0.224	4.457	12
41	Noodles with bontux sauce	0.575	1.818	72.076	0.085	607.363	0.921	0.236	32.618	12
42	Tomatican with rice	0.497	0.917	2.804	0.225	1354.996	8.092	0.596	7.632	12
43	Minestrone	4.947	4.666	251.471	0.054	595.842	1.465	0.064	31.541	14
44	Roasted chicken with mixed mash	0.284	0.384	36.214	0.083	685.767	1.043	0.201	2.415	11
45	Beef stew with whole rice	0.534	1.219	2.598	0.201	1116.777	6.108	0.522	3.661	11
46	Pork stew with rice	4.690	1.684	77.280	0.189	1077.586	2.222	-0.186	5.175	10
47	Beef roast with creamed corn	1.391	4.786	72.947	0.199	1915.906	5.793	0.778	53.113	9
48	Pork stew with au gratin potatoes	4.724	1.300	8.010	0.207	1213.846	2.471	-0.314	3.577	14
49	Pea and pork cutlet stew with curry rice	1.393	1.634	74.252	0.186	1057.762	1.679	-0.378	2.559	12
50	Chicken and green peas stew with rice noodles	1.384	4.685	38.370	0.093	694.807	1.284	0.301	30.297	11
51	Beef fajitas x2	0.557	1.068	37.260	0.210	1714.443	5.248	0.342	8.343	10
52	Beef stew with mixed mash	3.917	1.649	7.086	0.219	1283.587	6.396	0.416	1.421	15
53	Oven-roasted turkey with spring rice	4.765	1.513	43.380	0.125	1072.225	0.945	0.446	4.576	12
54	Roasted chicken with quinoa	0.852	3.855	3.468	0.139	1171.969	1.137	0.488	1.743	10
55	Roasted chicken with creamed spinach	0.889	2.669	3.927	0.136	1131.226	1.799	0.349	22.581	12
56	Vegetable pudding with quinoa	0.973	2.161	39.002	0.044	713.539	1.144	0.317	55.319	15
57	Roasted turkey with vegetable medley	0.294	1.157	35.966	0.138	1459.590	1.774	0.466	1.998	15
58	Zucchini squash stew with rice	0.771	0.678	8.886	0.103	990.521	3.382	0.389	56.928	12
59	Meat in mushroom sauce with noodles	1.997	3.937	38.064	0.262	1450.111	6.542	0.722	29.647	13
60	Beans with pilco	5.645	1.289	10.026	0.024	582.471	0.750	-0.144	22.673	12
61	Chicken lasagna with vegetables	0.177	0.284	1.313	0.119	987.738	2.118	0.206	35.795	18
62	Roasted meat with vegetable medley	0.807	1.649	73.181	0.232	1411.306	6.977	0.647	21.102	16
63	Poblano cream with breaded steak	5.547	1.309	44.094	0.218	1839.641	5.300	0.386	4.329	9
64	Veracruz-style fish with vegetable rice	4.004	1.792	7.792	0.271	1288.950	1.644	0.571	0	5
65	Mexican flautas	1.349	3.609	1.798	0.149	1397.312	1.787	-0.386	0	6
66	Sausage and bean stew	0.869	1.441	4.147	0.104	1270.311	2.399	-0.263	35.577	13
67	Ratatouille	0.848	0.971	82.348	0.139	1354.666	10.471	0.057	29.848	13
68	Vegetable curry with rice	0.784	1.628	772.432	0.128	857.754	3.015	0.360	36.910	12
69	Vegetable lasagna	0.831	3.843	55.756	0.199	1843.961	4.141	0.265	65.381	13

Table S5. *(continued)*

N°	Main courses	Environmental			Economic		Social		Political (%)	Techno-logical
		GWP (CO2 eq)	Water use (m3)	Species.yr	USD2013	Cost ($)	DALY	NRF 9.3		
70	Quinoa risotto	1.438	1.493	113.979	0.111	1546.035	1.362	0.315	32.203	6
71	Spinach risotto	0.623	2.299	4.156	0.033	381.764	0.715	0.221	24.632	8
72	Vegetarian corn pie	0.366	4.445	0.799	0.255	4191.175	5.670	0.092	65.412	12
73	Roasted pulp with creamed chard	3.007	2.047	7.044	0.336	1823.482	2.986	-0.890	30.074	12
74	Vegetarian meat and vegetable stew	2.330	2.578	41.371	0.051	581.551	1.752	0.130	20.085	12
75	Vegetarian potato pie	0.392	0.926	36.464	0.061	612.913	1.098	0.009	17.259	14
76	Pork pulp with vegetable medley	0.459	0.394	2.013	0.331	1881.776	2.984	-0.758	17.546	14
77	Arab rice with goulash	2.261	2.772	40.973	0.209	1120.966	6.649	0.657	14.869	13
78	Noodles with bolognese sauce	4.761	3.090	112.157	0.208	1099.623	5.576	0.329	35.413	12
79	Roasted chicken with spring rice	4.391	0.923	42.382	0.121	903.974	1.288	0.361	5.036	10
80	Vegetarian tomatican with rice	0.906	4.566	3.343	0.067	670.261	3.520	0.201	24.643	13
81	Oven-baked pork pulp with mash	0.575	3.303	244.733	0.345	1919.077	2.918	-0.869	1.439	13
82	Oven-baked fish with rice	2.346	2.659	41.684	0.205	1060.091	1.144	0.217	2.166	10
83	Vegetarian charquican	1.099	2.941	36.345	0.050	543.841	1.447	0.152	25.455	13
84	Stuffed zucchini with quinoa	0.332	0.612	1.420	0.208	1652.085	6.850	0.560	40.521	11
85	Beef stew with mash	4.435	2.042	112.036	0.314	1752.131	9.663	0.738	4.646	13
86	Vegetable pudding with rice	7.427	1.762	117.061	0.051	531.483	2.244	-0.043	41.438	14
87	Swiss chard stew with roasted potatoes	0.578	3.234	106.037	0.058	577.951	1.593	-0.198	27.829	13
88	Pork and green peas stew with noodles	0.338	0.482	36.332	0.365	2055.597	3.297	-0.452	26.082	13
89	Roasted chicken with vegetable medley	2.377	2.591	41.650	0.130	1081.036	1.817	0.379	0.276	10
90	Noodles with mushroom and corn sauce	0.829	1.920	3.706	0.067	843.893	1.276	0.108	58.954	15
91	Vegetarian pantrucas	0.361	0.469	2.045	0.054	509.957	1.693	0.038	51.147	15
92	Beef with mushroom and au gratin potatoes	0.385	0.403	70.791	0.238	1387.616	6.377	0.518	0.428	12

Table S6. Evaluation of Sustainability Dimensions - Desserts. GWP: Global Warming Potential, NRF 9.3: Nutrient Rich Foods Index.

N°	Dessert	Environmental			Economic		Social		Political (%)	Techno-logical
		GWP (CO2 eq)	Water use (m3)	Species.yr	USD2013	Cost ($)	DALY	NRF 9.3		
1	Apple	0.041	-0.018	0.262	0.007	178.640	0.542	0.075	0	1
2	Semolina pudding	0.412	0.461	2.809	0.054	266.094	0.609	-0.045	0	4
3	Orange	0.071	0.744	0.184	0.006	128.000	0.645	0.110	100	1
4	Fruit jelly	0.022	0.108	0.144	0.005	34.662	0.079	0.011	0	2
5	Pear	0.077	0.427	0.291	0.010	151.920	0.543	0.076	0	1
6	Pineapple	0.072	0.084	0.263	0.008	143.040	0.653	0.071	0	1
7	Brazo de reina	0.118	0.242	0.362	0.015	123.931	0.200	0.037	33	4
8	Roasted milk	0.106	0.073	0.318	0.010	86.447	0.140	-0.005	41	3
9	Bavarian cream	0.035	0.079	0.235	0.009	196.220	0.137	0.001	0	2
10	Chocolate flan	0.749	0.325	1.700	0.052	732.127	0.730	-0.021	33	5
11	Carrot cake	0.151	0.362	0.640	0.018	224.515	0.222	-0.218	50	7
12	Vanilla flan	0.459	0.234	1.117	0.045	652.127	0.607	-0.032	29	4
13	Caramel flan	0.459	0.234	1.117	0.045	652.127	0.607	-0.032	29	4
14	Raspberry mousse	0.056	0.161	0.354	0.014	234.970	0.203	0.004	0	3
15	Pineapple jelly	0.015	0.052	0.075	0.003	32.712	0.077	0.005	0	2
16	Wheat with dried peaches	0.111	1.040	0.588	0.017	166.615	0.401	0.020	0	3
17	Marble jelly	0.075	0.109	0.502	0.012	100.432	0.114	-0.094	0	2
18	Fried bananas with cream	0.723	2.547	5.189	0.140	836.466	1.991	-0.022	0	4
19	Tricolor jellies	0.070	0.177	0.293	0.013	125.233	0.159	-0.003	0	2
20	Candied pumpkin	0.033	0.210	0.129	0.005	85.820	0.313	0.024	0	1
21	Peach	0.075	0.491	0.283	0.010	150.600	0.541	0.061	0	1
22	Strawberry	0.095	0.956	0.423	0.019	363.000	0.597	0.069	0	1
23	Apricot	0.062	0.376	0.266	0.007	216.000	0.502	0.086	0	1
24	Cherry	0.114	4.631	1.059	0.026	257.640	0.598	0.053	0	1
25	Kiwi	0.104	0.350	0.301	0.019	171.600	0.567	0.072	0	1
26	Tangerine	0.117	0.904	0.514	0.018	141.720	0.624	0.078	0	1
27	Papaya	0.070	0.218	0.310	0.010	478.800	0.493	0.072	0	1
28	Banana	0.139	0.740	1.022	0.029	130.680	0.513	0.123	0	1
29	Mango	0.136	1.398	1.056	0.030	285.120	0.547	0.077	0	1
30	Raspberry	0.154	0.978	1.198	0.040	312.000	0.660	0.201	0	1
31	Fig	0.065	2.263	0.320	0.010	718.800	0.657	0.107	100	1
32	Melon	0.037	0.121	0.110	0.004	73.867	0.326	0.035	0	1
33	Watermelon	0.049	0.463	0.274	0.012	74.880	0.393	0.018	0	1

Table S7. Menus. EW: Equitable Weighting; BW: Biased Weighting.

N°	Starter	Main course	Dessert	Ranking Indicators		Ranking Dimensions	
				EW	BW	EW	BW
1	Lettuce with carrot	Lentil stew	Orange	4	4	58	58
2	Tomatoes with green beans	Vegetarian corn pie	Pear	53	53	1	1
3	Cabbage mix	Roast beef with creamed swiss chard	Jelly with fruit	10	10	10	10
4	Lettuce with carrot	Noodles with vegetables and sauce	Apple	32	32	9	9
5	”Empanada de pino”	Vegetarian carbonara	Tangerine	60	60	8	8
6	Tomato with onion	Beans with noodles	Pear	61	61	45	45
7	Chicken bouillon	Roast chicken with spring vegetable rice	Tangerine	21	21	41	41
8	Tomato with onion	Beans with corn	Apple	43	43	56	56
9	Lettuce with green beans and carrot	Noodles with sauce bontux	Pear	49	49	30	20
10	Cabbage and lettuce mix	Vegetarian potato pie	Orange	1	1	48	67
11	Beetroot with carrot and coriander	Roast pork with mixed vegetables	Raspberry mousse	41	41	20	30
12	Beef bouillon	Goulash with arabic rice	Banana	28	28	67	48
13	Cabbage with beetroot	Beef with noodles	Jelly with fruit	13	13	4	4
14	Tomatoes with coriander	Baked fish with mashed potatoes	Apple	58	55	21	21
15	Vegetable cream	Pork with quinoa	Flan with caramel	55	58	66	18
16	”empanada de pino”	Beef grilled with tomato and onion	Apple	31	31	61	61
17	Beetroot with carrot	Beef roasted with vegetable stews	Pineapple	17	65	18	66
18	Cabbage mix with corn	Chicken soup	Roasted milk	65	17	33	36
19	Vegetable cream	Roast turkey with rice and vegetables	Chocolate flan	5	5	36	33
20	Lettuce with spinach and carrot	Chicken roasted with quinoa	Orange	40	40	53	53
21	Celery with carrot	Noodles with vegetables and sauce	Pineapple	63	63	59	59
22	Tomatoes with coriander	Chickpeas stew	Apple	20	20	14	51
23	Lettuce with spinach	Chicken with green beans and rice	Marble jelly	27	27	23	23
24	Beef bouillon	Beef with sweet corn cream	Pear	42	42	62	62
25	Noodles soup	Scalopino milanese with cream	Fried bananas with cream	59	59	6	22
26	Tomato with lettuce	Fish with rice and vegetables	Jelly	36	36	22	6
27	Carrot with cucumber	Mexican tacos	Pumpkin with cinnamon	9	9	50	50
28	Vegetable cream	Stroganoff with rice	Pear	64	64	51	43
29	Lettuce with mixed bell peppers	Beef soup	Roasted milk	18	35	37	14
30	Tomato with onion	Lentil stew	Pineapple	35	18	52	7
31	Vegetable cream	Stroganoff with rice	Jelly with fruit	51	47	7	52
32	Beetroot with coriander	Carbonada	Apple	47	51	43	32
33	Lettuce with green beans and carrot	Noodles with sauce bontux	Semolina pudding	29	29	32	65
34	Pumpkin cream	Beef fajitas x2	Peach	46	46	26	37
35	Tomato with cucumber	Beef with mashed potatoes	Apple	34	34	65	27
36	Vegetable cream	Roast turkey with rice and vegetables	Roasted milk	8	8	38	38
37	Celery with carrot and olives	Chicken roasted with quinoa	Flan with caramel	66	66	27	26
38	Chicken bouillon	Chicken roasted with creamed spinach	Peach	19	19	42	60
39	Tomatoes with green beans	Chicken with green beans and mashed potatoes	Marble jelly	11	11	19	39
40	Lettuce with tuna	Tomatican with rice	Strawberry	15	14	5	5
41	Pumpkin cream	Vegetables pudding with quinoa	Roasted milk	14	15	60	19
42	Pizza	Roast turkey with vegetables	Apricot	2	2	39	13
43	Cabbage mix	Zucchini stew with rice	Pineapple	67	67	13	42
44	Artichoke	Beef with noodles and mushroom sauce	Jelly with fruit	54	12	44	17
45	Tomatoes with coriander	Beans with corn	Pear	12	54	17	44
46	Lettuce with spinach	Chicken lasagne with vegetables	Vanilla flan	33	33	46	46
47	Chicken bouillon	Beef fajitas x2	Peach	44	44	57	57
48	Tomatoes with coriander	Lentil stew	Pear	56	25	24	55
49	Hydroponic lettuce with carrot and peas	Pasta with bolognese sauce	Vanilla flan	52	52	55	24
50	Chicken bouillon	Roast chicken with spring vegetable rice	Pear	25	56	49	49
51	Lettuce with carrot	Vegetarian tomatillo with rice	Orange	23	23	34	63
52	Cabbage and olive	Chicken with green beans and rice	Jelly with fruit	6	57	63	29
53	Tomato with onion	Minestrone	Apple	45	45	47	34
54	Cabbage and lettuce mix	Roast beef with mashed potatoes	Roasted milk	57	6	29	47
55	Celery with corn	Pasta with bolognese sauce	Tangerine	24	24	35	35
56	Tomato with onion	Beans with corn	Pear	16	16	16	2
57	Celery with cabbage	Baked fish with rice	Raspberry mousse	3	3	2	16
58	Lettuce with green beans and carrot	Vegetarian charquican	Orange	62	62	64	64
59	Beetroot with carrot	Chicken roasted with creamed spinach	Tangerine	38	38	31	31
60	Celery with corn	Zucchini stew with rice	Apple	37	37	12	28
61	Lettuce with carrot	Noodles with vegetables and sauce	Pineapple	30	30	28	12
62	Tomatoes with coriander	Beans with noodles	Pear	48	48	15	15
63	Pumpkin cream	Stuffed zucchinis with quinoa	Apple	50	50	25	25
64	Celery with corn	Beef with mashed potatoes	Tangerine	7	7	40	40
65	Tomatoes with coriander	Beans grits	Pear	22	22	3	3
66	Lettuce with green beans and carrot	Noodles with sauce bontux	Vanilla flan	39	39	11	11
67	Cabbage mix with corn	Vegetarian carbonara	Banana	26	26	54	54

Table S8. Ranking Starter. EW: Equitable Weighting; BW: Biased Weighting.

N°	Starter	Ranking Indicators		Ranking Dimensions		N°	Starter	Ranking Indicators		Ranking Dimensions	
		EW	BW	EW	BW			EW	BW	EW	BW
1	Beetroot with onion and carrot	53	53	11	11	29	Cabbage mix with corn	36	54	1	17
2	Red cabbage with peppers	5	5	13	13	30	Celery with carrot	54	23	54	43
3	Celery with avocado and other vegetables	11	11	10	10	31	Noodle soup	43	36	51	45
4	Lettuce with broad beans	44	44	8	20	32	Tomato with lettuce	38	38	42	54
5	Cream of vegetable soup	10	10	20	26	33	Carrot with cucumber	52	29	43	51
6	Tomato with coriander	26	26	26	8	34	Lettuce with green beans and carrot	29	52	45	35
7	Beef consomme	25	25	29	29	35	Cream of pumpkin soup	24	24	35	42
8	Cabbage mix	20	20	27	28	36	Celery with carrot and olive	18	18	5	5
9	Lettuce with corn	1	1	28	21	37	Lettuce with spinach and carrot	4	35	32	4
10	Beetroot with coriander	7	45	56	14	38	Lettuce with tuna	35	4	4	53
11	Spinach with carrot	45	7	14	27	39	Vegetarian pizza	42	42	53	32
12	Tomato with green bean	13	13	21	56	40	Pizza	40	40	48	48
13	Broccoli with cauliflower	21	21	30	15	41	Artichokes	50	50	38	38
14	Lettuce with spinach	39	39	37	30	42	Spinach with celery and corn	51	51	49	49
15	Poultry consomme	56	56	15	37	43	Cabbage mix with lettuce	48	48	39	39
16	Tomato with cucumber	19	19	9	9	44	Beetroot with carrot and coriander	49	15	50	50
17	Lettuce with carrot	8	14	23	44	45	Lettuce with carrot and peas	15	49	52	16
18	Celery with olives	14	8	19	23	46	Carrot with spinach and spring	12	2	16	12
19	Lettuce with spring salad	37	37	18	41	47	Tomato with onion	2	12	12	52
20	Beetroot with carrot	17	17	31	31	48	Celery with corn	31	31	6	6
21	Green beans with corn	30	27	44	18	49	Celery with cabbage	41	41	40	40
22	Lettuce with bell pepper mix	27	30	41	34	50	Cabbage mix with olives	22	22	47	47
23	Celery, spinach and olives	28	28	36	19	51	Cabbage with corn and celery	32	32	22	22
24	Lettuce with mushroom	34	9	46	46	52	Lettuce with celery and carrot	47	47	2	2
25	Meat pie	33	34	34	33	53	Cabbage with olives	16	16	7	7
26	Beetroot with onion	9	33	24	1	54	Lettuce with carrot and corn	3	3	25	25
27	Celery with spinach	46	46	33	36	55	Celery with peppers	6	6	3	3
28	Cabbage with beetroot	23	43	17	24	56	Spinach, celery with corn and olives	55	55	55	55

Table S9. Ranking Main courses. EW: Equitable Weighting; BW: Biased Weighting.

N°	Main course	Ranking Indicators		Ranking Dimensions		N°	Main course	Ranking Indicators		Ranking Dimensions	
		EW	BW	EW	BW			EW	BW	EW	BW
1	Roasted turkey with creamed spinach	90	90	60	60	47	Beef roast with creamed corn	20	45	19	35
2	Beef stew with rice	87	87	33	71	48	Pork stew with au gratin potatoes	45	20	80	19
3	Charquican	91	91	71	33	49	Pea and pork cutlet stew with curry rice	35	35	38	39
4	Chicken stewed with quinoa	36	36	83	83	50	Chicken and green peas stew with rice noodles	74	46	61	1
5	Beef stew with noodles	8	58	15	41	51	Beef fajitas x2	46	74	39	61
6	Chicken casserole	58	8	41	15	52	Beef stew with mixed mash	14	14	78	78
7	Chilean bean soup with spaghetti	56	56	90	90	53	Oven-roasted turkey with spring rice	2	2	82	23
8	Vegetable pudding with boiled potatoes	59	59	56	56	54	Roasted chicken with quinoa	52	52	5	5
9	Noodles with seafood sauce	33	33	75	75	55	Roasted chicken with creamed spinach	82	82	23	82
10	Stewed chickpeas	40	40	9	43	56	Vegetable pudding with quinoa	1	1	29	29
11	Meat and vegetable stew	70	70	43	9	57	Roasted turkey with vegetable medley	26	43	59	59
12	Pork stew with quinoa	75	75	44	50	58	Zucchini squash stew with rice	43	26	3	66
13	Stroganoff with rice	78	78	50	91	59	Meat in mushroom sauce with noodles	92	25	66	69
14	Baked fish with mashed potatoes	30	30	91	44	60	Beans with pilco	6	6	69	3
15	Stewed lentils	84	84	74	74	61	Chicken lasagna with vegetables	25	92	11	84
16	Chicken and green peas stew with mashed potatoes	69	69	7	7	62	Roasted meat with vegetable medley	85	85	28	11
17	Chicken stew with rice	86	86	36	36	63	Poblano cream with breaded steak	65	65	65	28
18	Valencian rice	29	29	70	8	64	Veracruz-style fish with vegetable rice	71	71	20	20
19	Pantrucas	42	42	34	34	65	Mexican flautas	41	41	51	65
20	Ajiaco	9	9	8	70	66	Sausage and bean stew	67	67	84	51
21	Chicken and green peas stew with rice	66	66	87	87	67	Ratatouille	10	10	26	26
22	Beef tenderloin with corn pudding	44	39	10	10	68	Vegetable curry with rice	80	18	63	45
23	Roasted meat with creamed spinach	5	5	30	30	69	Vegetable lasagna	18	53	45	63
24	Oven-roasted ribs with vegetable medley	39	44	53	53	70	Quinoa risotto	88	38	62	77
25	Chicken stew with rice	60	22	54	79	71	Spinach risotto	53	80	92	62
26	Beef casserole	50	50	14	54	72	Vegetarian corn pie	38	88	32	67
27	Corn pie	22	60	79	89	73	Roasted pulp with creamed chard	4	34	77	92
28	Mixed grill with chilean salad	11	11	89	21	74	Vegetarian meat and vegetable stew	89	4	67	32
29	Potato pie	51	51	31	14	75	Vegetarian potato pie	34	89	72	72
30	Beans with mazamorra	7	7	57	16	76	Pork pulp with vegetable medley	73	21	2	2
31	Seafood soup	63	63	21	6	77	Arab rice with goulash	54	54	52	27
32	Beef with coriander and vegetable medley	62	62	6	31	78	Noodles with bolognese sauce	21	73	27	12
33	Humitas x2	83	19	64	18	79	Roasted chicken with spring rice	37	37	12	46
34	Roast chicken with rice	19	83	40	86	80	Vegetarian tomatican with rice	48	48	85	52
35	Oven-baked fish with spring quinoa	32	32	16	40	81	Oven-baked pork pulp with mash	17	17	42	42
36	Noodles with spring sauce	23	3	4	68	82	Oven-baked fish with rice	16	16	46	85
37	Pork stew with au gratin potatoes	3	23	18	25	83	Vegetarian charquican	68	68	49	49
38	Chicken stew with spring rice	61	61	55	58	84	Stuffed zucchini with quinoa	12	12	48	48
39	Zucchini squash stew with roasted potatoes	77	13	35	55	85	Beef stew with mash	24	24	13	13
40	Chicken stew with quinoa	57	77	47	4	86	Vegetable pudding with rice	47	47	37	37
41	Noodles with bontux sauce	13	57	25	17	87	Swiss chard stew with roasted potatoes	79	79	24	24
42	Tomatican with rice	15	72	68	47	88	Pork and green peas stew with noodles	76	76	88	88
43	Minestrone	72	27	86	64	89	Roasted chicken with vegetable medley	49	49	73	73
44	Roasted chicken with mixed mash	31	15	17	57	90	Noodles with mushroom and corn sauce	64	64	76	76
45	Beef stew with whole rice	27	31	58	80	91	Vegetarian pantrucas	55	55	81	81
46	Pork stew with rice	28	28	1	38	92	Beef with mushroom and au gratin potatoes	81	81	22	22

Table S10. Ranking Desserts. EW: Equitable Weighting; BW: Biased Weighting.

N°	Desserts	Ranking Indicators		Ranking Dimensions		N°	Desserts	Ranking Indicators		Ranking Dimensions	
		EW	BW	EW	BW			EW	BW	EW	BW
1	Apple	15	15	15	15	18	Fried bananas with cream	31	31	25	7
2	Semolina pudding	8	8	4	32	19	Tricolor jellies	27	27	14	26
3	Orange	7	7	32	4	20	Candied pumpkin	25	25	26	28
4	Fruit jelly	4	4	20	3	21	Peach	33	33	28	14
5	Pear	2	2	3	20	22	Strawberry	23	23	17	17
6	Pineapple	11	11	6	6	23	Apricot	18	18	22	22
7	Brazo de reina	9	9	9	23	24	Cherry	5	5	30	30
8	Roasted milk	12	12	8	9	25	Kiwi	21	21	29	29
9	Bavarian cream	13	13	23	8	26	Tangerine	22	22	16	24
10	Chocolate flan	10	10	5	5	27	Papaya	26	26	24	16
11	Carrot cake	14	14	27	33	28	Banana	16	16	12	1
12	Vanilla flan	17	17	33	31	29	Mango	28	28	13	12
13	Caramel flan	6	6	31	21	30	Raspberry	30	30	11	13
14	Raspberry mousse	32	32	21	27	31	Fig	29	29	10	11
15	Pineapple jelly	20	20	2	2	32	Melon	24	24	1	10
16	Wheat with dried peaches	19	19	19	25	33	Watermelon	1	1	18	18
17	Marble jelly	3	3	7	19						
